# α1A Adrenoreceptor blockade attenuates myocardial infarction by modulating the integrin-linked kinase/TGF-β/Smad signaling pathways

**DOI:** 10.1186/s12872-023-03188-w

**Published:** 2023-03-24

**Authors:** Nawal M. Alrasheed, Raghad B. Alammari, Tahani K. Alshammari, Maha A. Alamin, Abeer O. Alharbi, Asma S. Alonazi, Anfal F. Bin Dayel, Nouf M. Alrasheed

**Affiliations:** 1 Department of Pharmacology and Toxicology, College of Pharmacy , King Saud University, P.O. Box 70474, Riyadh, 11567 Saudi Arabia; 2 Pharm D. Student, College of Pharmacy, King Saud University, Riyadh, Saudi Arabia

**Keywords:** Tamsulosin, Myocardial infarction, Integrin-linked kinase, Fibrosis, Isoproterenol

## Abstract

**Background:**

Myocardial infarction (MI) is considered a public health problem. According to the World Health Organization, MI is a leading cause of death and comorbidities worldwide. Activation of the α1A adrenergic receptor is a contributing factor to the development of MI. Tamsulosin, an α1A adrenergic blocker, has gained wide popularity as a medication for the treatment of benign prostatic hyperplasia. Limited evidence from previous studies has revealed the potential cardioprotective effects of tamsulosin, as its inhibitory effect on the α1A adrenoceptor protects the heart by acting on the smooth muscle of blood vessels, which results in hypotension; however, its effect on the infarcted heart is still unclear. The mechanisms of the expected cardioprotective effects mediated by tamsulosin are not yet understood. Transforming growth factor-beta (TGF-β), a mediator of fibrosis, is considered an attractive therapeutic target for remodeling after MI. The role of α1A adrenoceptor inhibition or its relationships with integrin-linked kinase (ILK) and TGF-β/small mothers against decapentaplegic (Smad) signaling pathways in attenuating MI are unclear. The present study was designed to investigate whether tamsulosin attenuates MI by modulating an ILK-related TGF-β/Smad pathway.

**Methods:**

Twenty-four adult male Wistar rats were randomly divided into 4 groups: control, ISO, TAM, and ISO + TAM. ISO (150 mg/kg, intraperitoneally) was injected on Days 20 and 21 to induce MI. Tamsulosin (0.8 mg/kg, orally) was administered for 21 days, prior to ISO injection for 2 consecutive days. Heart-to-body weight ratios and cardiac and fibrotic biomarker levels were subsequently determined. ILK, TGF-β1, p-Smad2/3, and collagen III protein expression levels were determined using biomolecular methods.

**Results:**

Tamsulosin significantly attenuated the relative heart-to-body weight index (*p* < 0.5) and creatine kinase-MB level (*p* < 0.01) compared with those in the ISO control group. While ISO resulted in superoxide anion production and enhanced oxidative damage, tamsulosin significantly prevented this damage through antioxidant defense mechanisms, increasing glutathione and superoxide dismutase levels (*p* < 0.05) and decreasing lipid peroxide oxidation levels (*p* < 0.01). The present data revealed that tamsulosin reduced TGF-β/p-Smad2/3 expression and enhanced ILK expression.

**Conclusion:**

Tamsulosin may exert a cardioprotective effect by modulating the ILK-related TGF-β/Smad signaling pathway. Thus, tamsulosin may be a useful therapeutic approach for preventing MI.

**Supplementary Information:**

The online version contains supplementary material available at 10.1186/s12872-023-03188-w.

## Introduction

Myocardial infarction (MI) is a leading cause of cardiovascular morbidity and mortality despite the control of risk factors, such as arteriosclerosis [1–4].

Since coronary diseases are the leading cause of death, the need for biomedical research to advance medical treatments for cardiovascular diseases is urgent [5]. Currently, cardiovascular-focused research has advanced the understanding of the underlying molecular processes and cell‒cell interactions that coordinate myocardial growth and fibrosis [6]. Myocardial fibrosis is controlled by numerous processes of fibrotic growth involving transforming growth factors (TGFs) [7–12].

Evidence indicates that increased expression of the downstream effectors of TGF-β signaling is associated with infarct healing. Although evidence also suggests that bioactive TGF-β is secreted in the cardiac extracellular matrix after infarction reperfusion, the mechanisms of TGF-β activation in the infarcted heart are poorly understood [13–15].

TGF-βs activate small mothers against decapentaplegic2/3 (Smad2/3) cascades and possibly activate Smad1 and Smad5 in certain cell types, providing an alternative Smad-dependent pathway for signal transduction [16, 17]. Taking into consideration the wide range of effects of Smad3 on all cell types, it is unclear whether the improved remodeling exhibited by mice with loss of Smad3 results from fibroblast-mediated actions. Therefore, it is crucial to investigate the role of Smad-dependent signaling in MI. In addition, previous studies have reported that matrix attachment is necessary for activation by growth factors, including TGF-β [17].

The process of fibrosis is also induced by the collaboration of TGF in addition to its receptors and extracellular matrix (ECM) receptors, such as integrins [11]. Integrins are transmembrane receptors that attach cells to the matrix and mediate outside-in signaling and inside-out signaling, controlling the activities of growth factor receptors, cytoplasmic kinases and ion channels [12].

The serine/threonine integrin-linked kinase (ILK) exerts a notable role in the integrin-actin interaction in addition to its structural and signaling roles in the function of integrins. Published reports indicate that ILK gene therapy dramatically improves cardiac function and attenuates ventricular remodeling in rat models of myocardial infarction, but it remains unknown whether, in the absence of underlying cardiac ischemia, ILK gene therapy improves cardiac performance in heart failure models [18].

Interestingly, recent data also demonstrate that α_1A_ adrenoceptors are expressed in only a subpopulation of myocytes. The most abundant documented transcript in the rodent heart is that of the α_1A_ adrenoceptor [19].

Tamsulosin is a third-generation α_1_-adrenergic antagonist used worldwide for the treatment of benign prostatic hyperplasia (BPH) [20]. Agents with preferential selectivity for the α_1A_ adrenoceptor, such as tamsulosin, have gained widespread popularity due to their lower incidence of cardiovascular side effects, resulting in a high safety profile; however, their cardioprotective effects require further investigation [21].

Although activation of the α_1A_ adrenoceptor can be a crucial factor in the development of MI, the relationship between ILK and MI, particularly the ability of an α_1A_ adrenoceptor blocker to attenuate MI via modulation of the ILK-related TGF-β/Smad pathway through the fibrotic pathway, still needs further clarification.

Therefore, we hypothesized that an α_1A_ adrenoceptor inhibitor might attenuate MI via modulation of ILK-related fibrosis (TGF-β/Smad) and that this proposed signaling cascade holds promise as a potential target for therapeutic intervention. The aims of our study were to first investigate the possible cardioprotective impact of tamsulosin against MI and to then explore the potential mechanisms by which tamsulosin mediates the attenuation of MI and determine whether this occurs via modulation of an ILK-related TGFβ/Smad signaling pathway.

## Material and methods

### Drugs, chemicals and antibodies

Tamsulosin and isoproterenol (ISO) were purchased from Sigma‒Aldrich (St Louis, MO, USA). The level of a cardiac biomarker (creatine kinase-MB (CK-MB)) was measured using specific ELISA kits for rats obtained from Cloud-Clone Corp. (Houston, Texas, US). Rat TGF-β was quantified using a Quantikine Rat TGF-β Immunoassay Kit (R&D Systems, Abingdon, UK) according to the manufacturer’s instructions. Anti-ILK, anti-TGF-β1, anti-Smad2/3, anti-collagen III and other related primary antibodies were purchased from Abcam (Biotechnology Inc., Cambridge, UK). Anti-rabbit and anti-mouse conjugated secondary antibodies were obtained from Sigma‒Aldrich (St. Louis, MO, USA). The ISO preparation was dissolved in distilled water and administered as a single subcutaneous bolus injection between the skin and underlying layers of tissue in the scapular region on the dorsal surface of each rat. Tamsulosin was dissolved in distilled water and administered orally via an oral gavage tube specific for Wistar rats. For all drugs used in this study, the doses were selected based on previously published studies, and the volume of each administered dose of each drug was calculated based on the rat’s body weight, which was measured prior to dosing, and administered in a 1 ml/kg dose volume. The efficacy and safety of tamsulosin (in the oral-controlled absorption system (OCAS®) formulation) have been assessed and improved on in several clinical trials.

### Experimental animals

Adult male Wistar rats weighing 150–250 g were supplied by the Animal Care Centre, College of Pharmacy, King Saud University. All animals were housed in a temperature-controlled room (23–25 °C) at 50% humidity and maintained on a 12-h light/dark cycle. Animals had free access to standard rat chow and water. The protocol of this study was performed in accordance with the National Institutes of Health Guidelines on the Care and Use of Laboratory Animals and was approved by the Animal Care Committee of King Saud University. The Research Ethics Committee approval number for this study is KSU-SE-19–61.

### Induction of acute myocardial infarction

For induction of myocardial infarction, ISO was dissolved in normal saline and injected intraperitoneally (*ip*; 150 mg/kg) on Days 20 and 21 at a 24-h interval [2]. Animals were euthanized 24 h after the second dose of ISO, at the end of Day 21.

### Experimental design

**Fig. 1:**
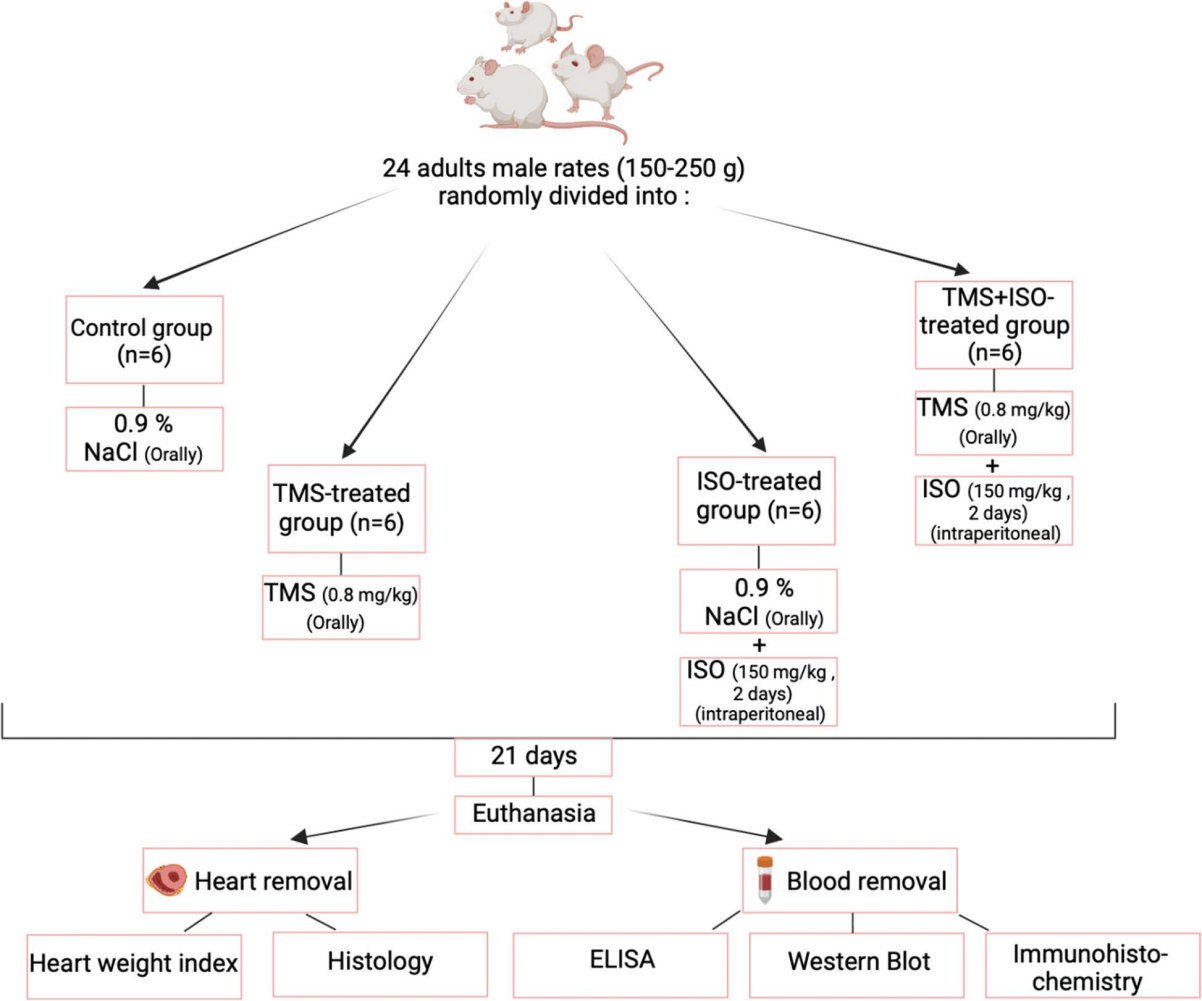
Flow chart demonstrating the experimental groups and design

Twenty-four rats were weighed and divided into four groups, each containing six rats (*n* = 6, Fig. 1). The rats were treated daily by oral gavage as follows for 21 continuous days:

#### Group 1

The control group; administered normal saline (0.9% NaCl) orally for 21 days.

#### Group 2

The ISO-treated group; treated with only normal saline for three weeks and then with ISO via injection (150 mg/kg) for two days (on the 20^th^ and 21^st^ days) [2].

#### Group 3

The tamsulosin-treated group; administered tamsulosin by oral gavage at a dosage of 0.8 mg/kg/day for 21 days [22].

#### Group 4

The tamsulosin + ISO-treated group; administered tamsulosin by oral gavage at a dosage of 0.8 mg/kg/day for three weeks and then treated with ISO via injection (150 mg/kg) for two days (on the 20^th^ and 21^st^ days).

At the end of the experiment, all rats were fasted overnight (12 h). Each rat was then weighed, anesthetized using a gradually increasing concentration of carbon dioxide (CO_2_) and sacrificed by decapitation. Blood samples were collected from nonsurviving experimental rats after anesthetization and sacrifice to expose of the animals to the least potential pain and stress. This terminal collection was accomplished via cardiac puncture, and the maximum amount of blood was collected. Then, the blood sample collected from each rat was processed to separate serum for assessment of cardiac and oxidative biomarkers using enzyme-linked immunosorbent assays (ELISAs) and biochemical assays, respectively. Hearts were excised, rinsed in ice-cold phosphate-buffered saline, and weighed. The heart weight-to-body weight ratio (HW/BW) was calculated as an index of MI. Cardiac samples were homogenized in cold phosphate-buffered saline (10% w/v), and clear homogenates were collected to assay fibrotic biomarkers using ELISA. Some heart tissues were fixed with neutral-buffered formalin (4%) for histological and immunohistochemical processing, while others were frozen at − 80 °C for subsequent molecular studies; samples from each rat in each group of 6 rats were collected and processed separately [23].

### Determination of cardiac biomarkers

The level of the cardiac biomarker CK-MB was quantified using serum samples and rat ELISA kits according to the specifications of the manufacturer. For the CK-MB assay, 100 μl of each standard or sample was incubated for 2 h at 37 °C. The wells were washed to remove any unbound substances, and then 100 μl of a conjugated antibody was added to each well and incubated for 1 h at 37 °C. Next, 200 μl of avidin-conjugated horseradish peroxidase (HRP) was added to all wells. The enzymatic reaction yielded a yellow-colored product; the intensity of the color was assessed by measuring the absorbance at 450 nm, and unknown concentrations of the samples were calculated using a standard curve.

### Enzyme immunosorbent assay (ELISA) of fibrosis markers

Levels of the fibrosis marker TGF-β1 were quantified using rat immunoassay ELISA kits. Homogenates or serum samples were placed into 96-well plates precoated with a monoclonal antibody specific for TGF-β1, and the assays were performed according to the manufacturer’s instructions.

### Assessment of oxidative stress biomarkers

Oxidative stress was estimated by measurement of glutathione (GSH), lipid peroxidation (LPO) and superoxide (SOD) in cardiac tissue homogenates. The GSH content was quantified using the method described by Moron et al. [24]. Briefly, 1 ml of heart homogenate was mixed with 1 ml of 25% trichloroacetic acid (TCA) and centrifuged at 4 °C and 3,000 rpm for 10 min. Then, 0.5 ml of the supernatant was added to 4.5 ml of Ellman’s reagent. The intensity of the resulting yellow color was measured spectrophotometrically at 412 nm and normalized to that of a reagent blank. GSH values are expressed as nmol per mg of protein in heart tissue. LPO was quantified using a thiobarbituric acid (TBA) assay as described by Ohkawa et al. [25]. Briefly, a mixture of 1 ml of 0.6% TBA, 2.5 ml of 20% trichloroacetic acid and 500 μl of heart homogenate was heated for 30 min in a boiling water bath, cooled, and centrifuged at 4 °C. The absorbance of the developed chromogen was measured at 535 nm and normalized to that of a reagent blank. The LPO values are expressed as nmol per mg of protein in heart tissue. SOD activity was estimated using a nitro blue tetrazolium method described by Delides et al. [26], and the degree of inhibition of the reaction was determined by spectrophotometric measurement at 430 nm. SOD activity is expressed as U mg^−1^ protein in heart tissue. One unit of SOD is defined as the amount of enzyme needed for dismutation of 50% of superoxide radicals.

### Western blot analysis

Western blotting was used to determine protein expression as previously described by Towbin et al. [27]. Briefly, frozen heart tissue samples were homogenized in ice-cold lysis buffer followed by immunoprecipitation assay (RIPA) buffer supplemented with an equal mixture of protease inhibitor and phosphatase inhibitor cocktails. Protein concentrations were determined using the Direct Detect quantification assay technique. Then, 60 μg of each homogenized protein sample was separated using sodium dodecyl sulfate‒polyacrylamide gel electrophoresis (SDS‒PAGE). The separated proteins on the gels were transferred to polyvinylidene difluoride (PVDF) membranes (0.2 μm, Immun-Blot®, Bio-Rad, California, USA). The membranes were incubated overnight at 4 °C with the primary antibodies, using a 1:1000 dilution ratio for the anti-ILK antibody. The β-actin antibody was used as a housekeeping loading control antibody and was diluted 1:2000. The membranes were washed and incubated for 1 h at room temperature with an HRP-conjugated anti-rabbit (1:5000) secondary antibody. Immunoblots were developed using an enhanced chemiluminescence (ECL) detection kit (GE Healthcare, Buckinghamshire, UK) for 2 min prior to image acquisition. Immunoreactive bands were visualized using an Image Quant LAS 4000 Mini imaging system (GE Healthcare, Buckinghamshire, UK). The intensities of the protein bands were densitometrically quantified using ImageJ software (NIH Image, Bethesda, MD, USA); bands of interest were normalized to a loading control (β-actin).

### Histological examination

Heart specimens were excised and immediately stored in 4% paraformaldehyde. Serial sections were cut at a 4 μm thickness using a Spencer 820 microtome and were used for histopathological examination after staining with hematoxylin and eosin (H&E). The sections were visualized at an original magnification of 10X, and the images were analyzed by an investigator specializing in this field.

### Immunohistochemistry

Fixed paraffin sections of heart tissue were used for the detection of collagen III and p-Smad2/3. Immunostaining was performed according to a method described in previous studies [17, 18] using the Immuno Cruz ABC staining system from Santa Cruz (Santa Cruz Biotechnology, Inc., California, USA). Nonspecific binding of antibodies was blocked by incubation with protein for 5 min. Sections were then incubated with the primary antibodies for 1 h at room temperature, washed three times in Tris buffer, and incubated with biotinylated anti-rabbit IgG (1:100 dilution) for 30 min. This was followed by washing, detection with a working solution of diaminobenzidine substrate, and finally staining with Mayer’s hematoxylin. The samples were analyzed using a bright field light microscope (DMRBE, Leica, Bensheim, Germany) equipped with a camera (ProgRes, Kontron Instruments, Watford, UK). The digital slide images were viewed at an original magnification of 10X and analyzed using Aperio`s viewing and image analysis tools.

### Statistical analysis

All data are expressed as the mean ± standard error of the mean (SEM) values, and statistical comparisons between groups were performed using one-way analysis of variance (ANOVA) followed by the Tukey‒Kramer post hoc test. Statistical analysis was conducted using GraphPad Prism software version 5 (GraphPad Software, Inc., San Diego, CA). A *P* value of ≤ 0.05 was considered statistically significant.

## Results

### Tamsulosin reduces the CK-MB content in myocardial tissue in response to ISO-induced cardiac damage

**Fig. 2:**
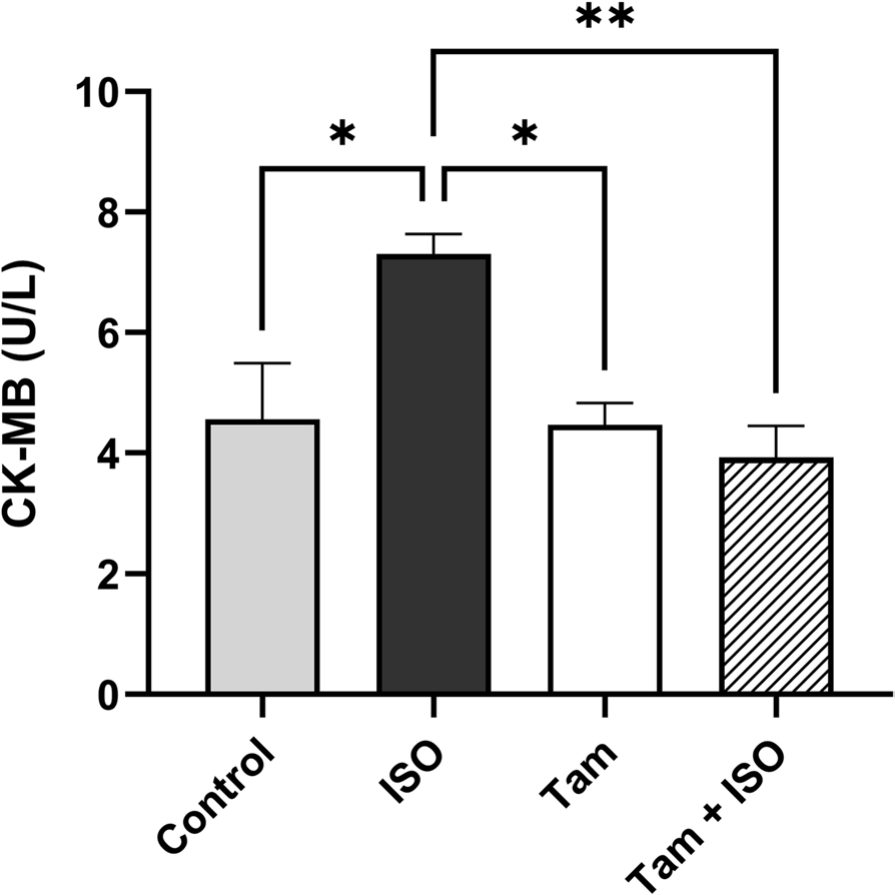
The effect of tamsulosin on the ISO-induced increase in CK-MB levels compared to those in the ISO-only and control groups. The data are expressed as the means ± SEMs (*n* = 6 duplicate samples from six different animals per group). Group comparisons were performed using one-way ANOVA followed by the Tukey‒Kramer post hoc test; *P < 0.05, **P < 0.01 compared to the ISO-only group; *P < 0.05 compared to the control group. Abbreviations: CK-MB, creatine kinase MB; ISO, isoproterenol; SEM, standard error of the mean; ANOVA, analysis of variance

As shown in Fig. 2, the CK-MB level was significantly increased in the ISO-only group compared to the control group (*P* < 0.05). However, administration of to ISO-treated rats significantly reduced the CK-MB level compared to that in rats in the ISO-only group (*P* < 0.01). Moreover, there was no significant difference in the CK-MB level between the control and tamsulosin groups. Previous studies with ISO as a model for MI indicated a very limited and minimal mortality rate, supporting the finding of our present study that the use of selected doses of ISO to induce MI was associated with minimal to negligible death rates.

### Tamsulosin inhibits isoproterenol-induced cardiac hypertrophy

As shown in Table 1, at the beginning of the experiment, there was a significant increase (*P* < 0.001) in the body weight in the ISO group compared to the control group. The body weight of rats treated with tamsulosin after ISO injection was significantly restored (*P* < 0.05) compared to that of those in the ISO-only group, while the body weight in the tamsulosin-only group did not differ significantly from that in the control group. Additionally, the body weights of the rats in all groups did not differ significantly at the end of the experiment.

**Table 1:** α_1A_ Adrenoceptor blockade reduces the body weight, heart weight and relative heart weight index in rats with ISO-induced MI

Group	Initial body weight (g)	Final body weight (g)	Heart weight (g)	Relative heart weight index (mg/g)
Control	139.00 ± 2.30	273.25 ± 5.60	0.95 ± 0.05	0.35 ± 0.02
ISO	267.67 ± 6.84***	237.00 ± 19.01	1.22 ± 0.04***	0.50 ± 0.30***
Tamsulosin	212.17 ± 22.45	265.00 ± 15.15	0.91 ± 0.02	0.36 ± 0.01**
ISO + Tamsulosin	207.67 ± 16.98^#^	278.75 ± 11.51	1.00 ± 0.07	0.43 ± 0.03^#^

The data are expressed as the mean ± SEM values (*n* = 6 rats per group). Group comparisons were performed using one-way ANOVA followed by the Tukey‒Kramer post hoc test; *P < 0.05, ***P < 0.001 compared to the control group; ^*#*^ *P* < 0.05 compared to the ISO-only group

*ISO* Isoproterenol, *SEM* Standard error of the mean, *ANOVA* Analysis of variance

Induction of ISO-mediated cardiac hypertrophy was assessed according to differences in the heart weight-to-body weight ratio among the treatment groups (Table 1). This ratio was significantly increased (*P* < 0.001) in the ISO-only group compared to the control group. The ratios in both the control and tamsulosin-only groups were significantly reduced (*P* < 0.05) compared to that in the ISO-only group. Treatment of ISO-induced MI in rats using tamsulosin slightly reduced the effect of cardiac injury on heart weight.

### Tamsulosin reduces the myocardial TGF-β level

**Fig. 3:**
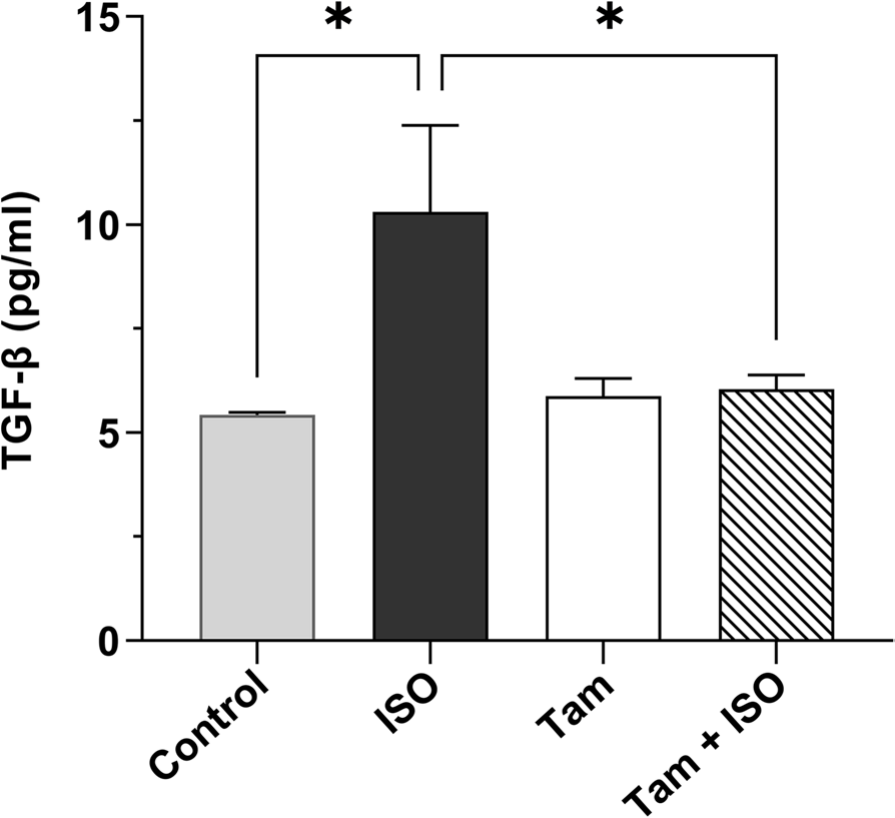
The effect of tamsulosin on ISO-induced increase in the TGF-β level. The data are expressed as the mean ± SEM values (*n* = 6 duplicate samples from six different animals per group). Group comparisons were performed using one-way ANOVA followed by the Tukey‒Kramer post hoc test; *P < 0.05 compared to the control and ISO-only groups. Abbreviations: ISO, isoproterenol; Tam, tamsulosin; TGF-β, transforming growth factor-beta; SEM, standard error of the mean; ANOVA, analysis of variance

As shown in Fig. 3, ISO-induced cardiac fibrosis was observed based on the high levels of the myocardial fibrosis marker TGF-β in the ISO-only group compared to the control group. The TGF-β level was significantly reduced (*P* < 0.05) after administration of tamsulosin to the ISO-treated rats compared to that in rats in the ISO-only group. Tamsulosin treatment alone did not significantly alter the cardiac TGF-β level compared to that in the control group.

### Tamsulosin attenuates experimentally induced cardiac hypertrophy through modulation of the ILK signaling pathway

**Fig. 4:**
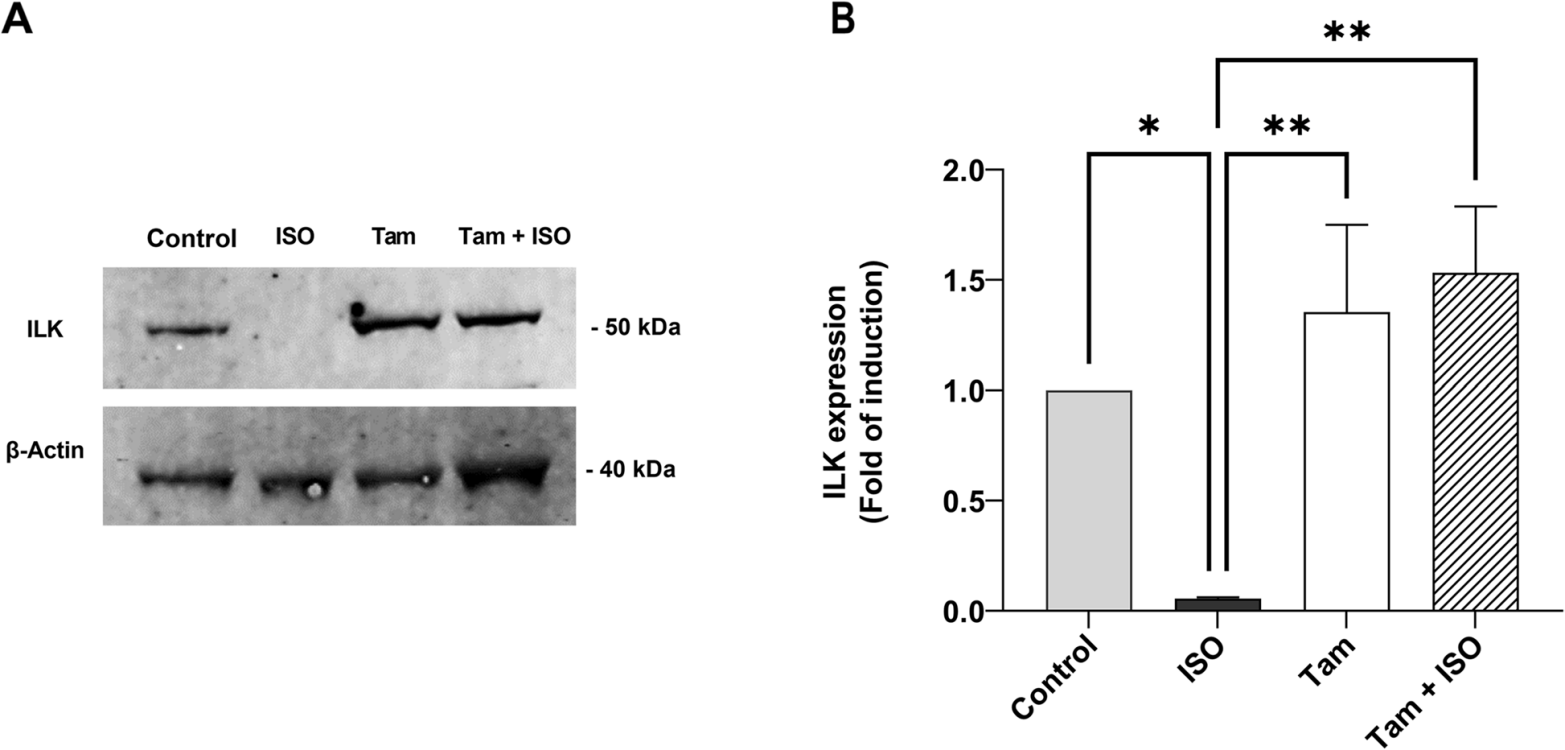
Tamsulosin modulates the ILK protein expression level in cardiomyocytes after MI. Analysis of the ISO-induced decrease in ILK expression in the ISO group compared to the other experimental groups by western blotting. (A). Representative immunoblot showing protein levels in tissue homogenates of the left ventricle in the control, isoproterenol-only (ISO), tamsulosin-only (Tam), and isoproterenol + tamsulosin (Tam + ISO) groups. Proteins in the tissue samples were separated by SDS‒PAGE and immunoblotted first with an anti-ILK antibody and then with an anti-β-actin antibody after stripping. The band densities on the immunoblots were quantified using ImageJ software. (B). Quantitative results of immunoblot analysis. ILK protein levels were normalized to those of β-actin. The quantities were expressed relative to the normal control group and expressed as fold induction values. The data are expressed as the mean ± SEM values (*n* = 6 samples from six different animals per group). Group comparisons were performed using one-way ANOVA followed by the Tukey‒Kramer post hoc test; *P < 0.05 compared to the control group; **P < 0.01 compared to the ISO-only group. Abbreviations: ISO, isoproterenol; tam, tamsulosin; ILK, integrin-linked kinase; SDS‒PAGE, sodium dodecyl sulfate‒polyacrylamide gel electrophoresis; SEM, standard error of the mean; ANOVA, analysis of variance

As shown in Fig. 4, the expression of the ILK protein in heart tissues of the treatment and control groups was quantified using western blotting (Fig. 4A). As shown in Fig. 3B, ISO-induced MI significantly decreased ILK expression in ISO-only group rats compared to the corresponding normal control group rats (*P* < 0.05). Remarkably, rats administered tamsulosin after experimental MI induction exhibited significantly increased levels of ILK expression, suggesting enhanced repair in cardiac cells, vessels, and muscle fibers (*P* < 0.01). Therefore, we examined ILK expression by western blotting continuously for four weeks following the injection of ISO. Figure 4A shows a higher immunoreactive band intensity indicating higher ILK expression in the hearts of the normal control and tamsulosin-treated groups than in the ISO-only group. Conversely, the ILK level was not altered in the myocardial tissue of normal rats treated with tamsulosin alone compared to rats in the normal control and ISO + tamsulosin groups.

### Tamsulosin reduces ISO-induced oxidative damage in the rat myocardium

As shown in Table 2, the LPO level was significantly increased in the ISO-only group (*P* < 0.05) compared to the control group. Treatment with tamsulosin alone reduced the LPO level compared to that in the ISO group, but this difference was not statistically significant. However, tamsulosin treatment after ISO administration resulted in a significant decrease in the LPO level compared to that in the ISO-only group (*P* < 0.001).

**Table 2:** The effects of tamsulosin on oxidative stress biomarkers in cardiac tissue from rats pretreated with ISO and control group rats

Group	LPO (nmol/mg)	GSH (nmol/mg)	SOD (U/mg)
Control	41.62 ± 3.44	9.77 ± 1.75	4.87 ± 1.21
ISO	70.79 ± 6.78*	4.80 ± 1.28*	0.588 ± 0.19*
Tamsulosin	52.62 ± 6.68	10.17 ± 1.11	5.31 ± 1.10
ISO + Tamsulosin	37.50 ± 6.80^*# # #*^	11.47 ± 1.16^*# #*^	4.23 ± 1.37^*#*^

The data are expressed as the mean ± SEM values (*n* = 6 duplicate samples from six different animals per group). Group comparisons were performed using one-way ANOVA followed by the Tukey‒Kramer post hoc test. *P < 0.05 compared to the control group; ^*#*^ *P* < 0.05, ^*##*^ *P* < 0.01, ^*###*^ *P* < 0.001 compared to the ISO-only group

*ISO* Isoproterenol, *LPO* Lipid peroxidation, *GSH* Glutathione, *SOD* Superoxide dismutase, *SEM* Standard error of the mean, *ANOVA* Analysis of variance

Using an ISO injection to induce MI significantly reduced the level of GSH in heart tissue compared to that in the control group (*P* < 0.05). Conversely, administration of tamsulosin to ISO-treated rats significantly increased the GSH level in heart tissue compared to that in the ISO-only group (*P* < 0.01). Additionally, administration of tamsulosin alone increased the GSH level compared to that in the ISO group.

Rats that received only ISO showed a significant reduction in cardiac SOD activity compared to that in the control group rats (*P* < 0.05). On the other hand, tamsulosin treatment after ISO administration significantly increased cardiac SOD activity (*P* < 0.05) compared to that in the ISO-only group.

### Tamsulosin reverses ISO-induced histopathological damage in rats in response to myocardial injury

**Fig. 5:**
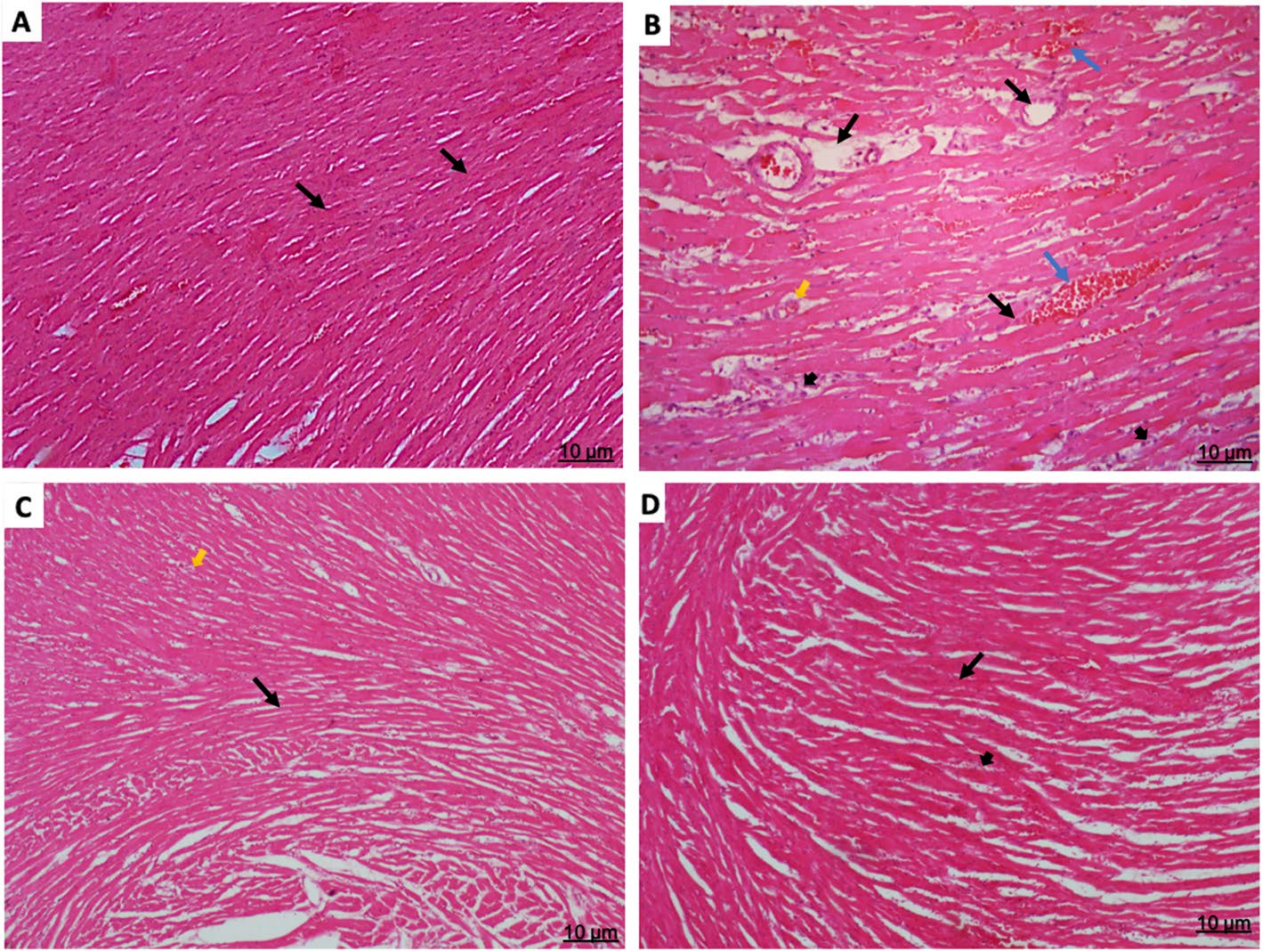
Histological photomicrographs of rat heart sections stained with H&E (scale bar 10 µm). **A** A section of a control heart showing normal myocardium fibers and cells within the normal heart vasculature. **B** ISO-treated rats had cardiomyocyte necrosis (black arrow) with greatly dilated, engorged blood vessels (blue arrow); increased edema (yellow arrow) in the intramuscular space; and massive inflammatory cell infiltration (small wide black arrow). **C** Tamsulosin-treated rats had relatively normal myocardial muscle fibers, fewer dilated blood vessels, and an edematous stroma. **D** A heart section from a tamsulosin + ISO group rat showing reduced myocardial degeneration and cellular infiltration with minimal infiltration of inflammatory cells. Abbreviation: ISO, isoproterenol; H&E, hematoxylin and eosin

As shown in Fig. 5, hematoxylin and eosin (H&E) staining revealed normal structures in the myocardial heart tissues, cells and fibers in control rats. In contrast, the myocardial heart tissues of rats in the ISO-only group exhibited severe inflammatory cell infiltration, extensive edema, necrosis, and greatly dilated, engorged blood vessels. Myocardial cells in the tamsulosin-only group were mostly preserved or only mildly damaged. Tamsulosin administration clearly ameliorated ISO-induced myocardial injury, as shown by the noticeable reductions in inflammatory cell infiltration and blood vessel degradation compared to rats in the ISO-only group.

### Tamsulosin prevents ISO-induced fibrosis and cardiac inflammation

**Fig. 6:**
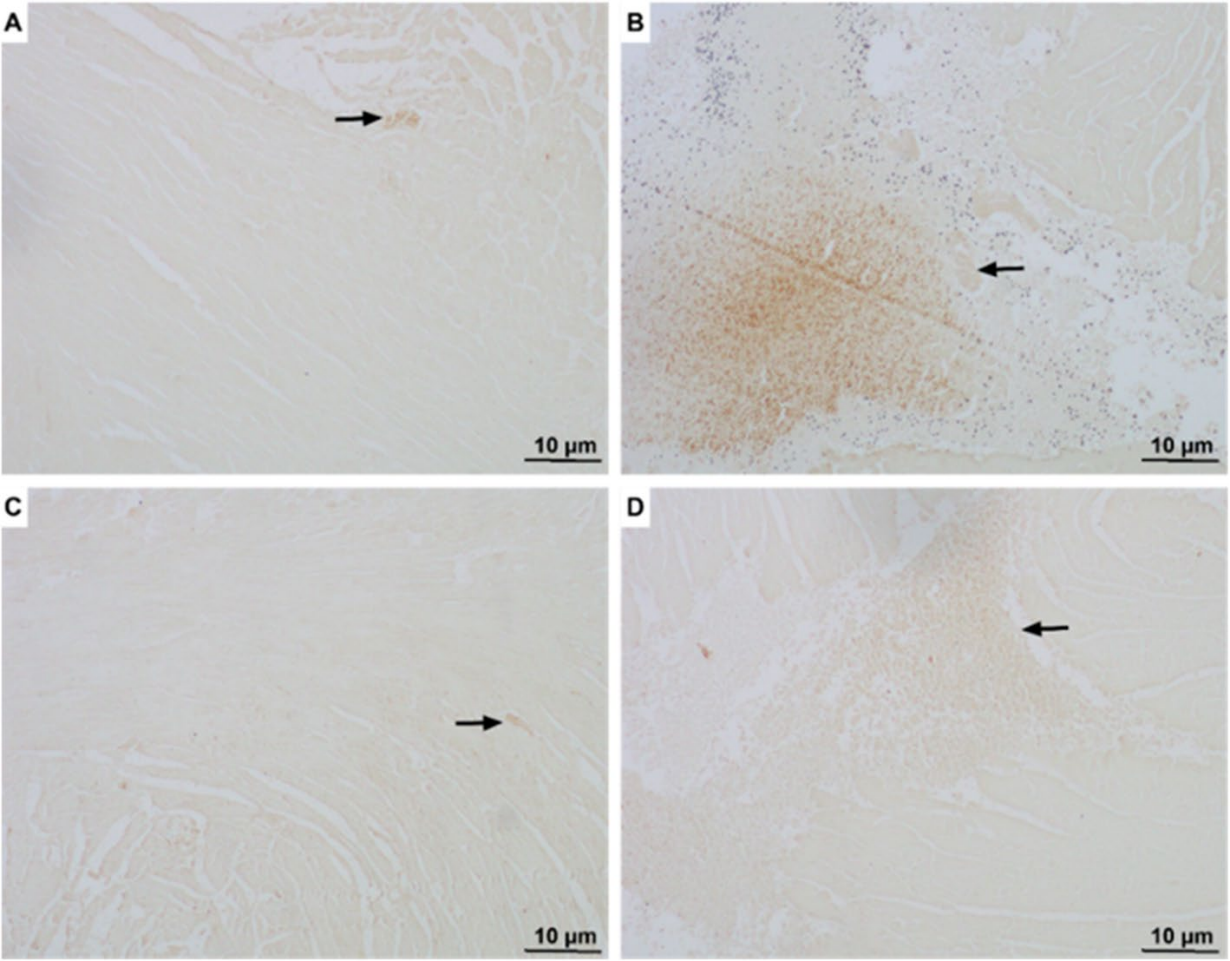
Effect of tamsulosin on ISO-induced collagen III expression in myocardial infarcts. **A** A representative section immunostained for collagen III (scale bar: 10 μm) showing normal collagen III deposition beside normal heart cells and vessels. **B** Administration of ISO alone increased the amount of collagen fibers and inflammation in the heart tissue (black arrow). **C** Animals treated with tamsulosin alone showed collagen III deposition similar to that in normal rats in the control group. **D** ISO-treated rats also treated with tamsulosin exhibited reduced interstitial collagen deposition, inflammation, and fibrosis (black arrow). Abbreviations: MI, myocardial infarction; ISO, isoproterenol

As shown in Fig. 6, immunohistochemical analysis revealed that ISO induced significant deposition of collagen III in the myocardium (Fig. 6B), which is an indication of fibrosis. Administration of tamsulosin to rats pretreated with ISO resulted in a lower level of collagen III compared to that in the ISO-only group (Fig. 6D). Consistent with this finding, collagen III deposition was minimal in the control group and tamsulosin-only group, as shown in Figs. 6A and C, respectively.

### Tamsulosin reduces p-Smad2/3 expression in myocardial infarcts in the hearts of ISO-treated rats

**Fig. 7:**
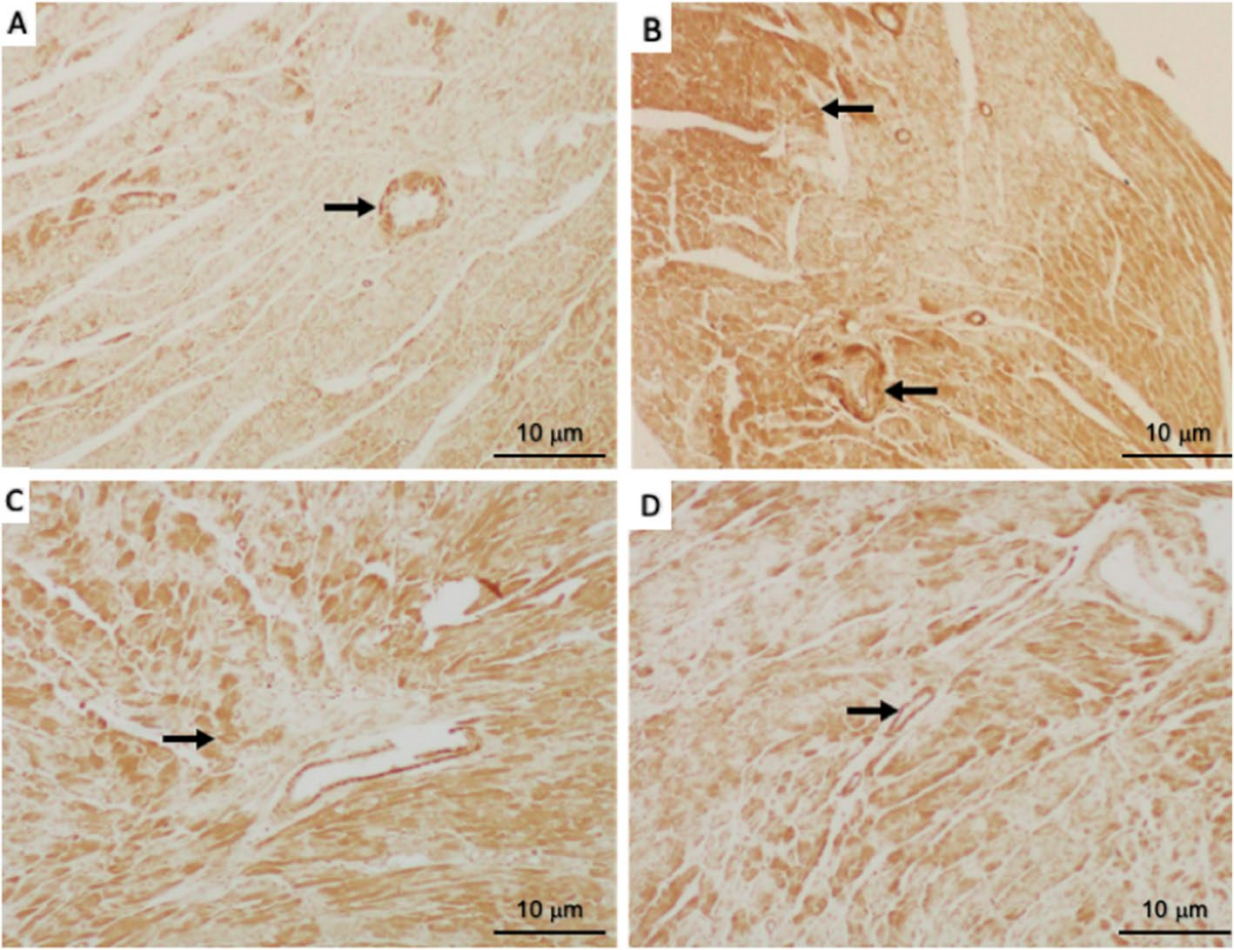
The effect of tamsulosin on Smad2/3 expression in the myocardial infarcts of ISO-treated rats. **A** Low levels of p-Smad2/3 in the myocardial and intracardiac vessels in the control group. **B** High levels of p-Smad2/3 in the heart vessels and large areas of fibrosis (black arrows) in the ISO-only group. **C** Treatment with tamsulosin alone decreased the levels of p-Smad2/3 and fibrosis in heart tissues. **D** Tamsulosin treatment after ISO administration significantly reduced fibrosis by reducing the p-Smad2/3 levels. Abbreviations: Smad2/3, small mothers against decapentaplegic2/3; ISO, isoproterenol

As shown in Fig. 7, immunohistochemical analysis of myocardium sections stained with an anti-p-Smad2/3 antibody revealed relatively low levels of endogenous p-Smad2/3 in control rats (Fig. 7A). ISO strongly increased the levels of P-Smad2/3 in cardiac vessels and elicited excessive fibrosis in tubules and cells (Fig. 7B). However, in the tamsulosin + ISO group, the levels of p-Smad2/3 were low, and there were fibrotic regions, indicating that tamsulosin treatment prevents damage by attenuating the levels of P-Smad2/3 expression levels in the setting of MI (Fig. 7D). Normal rats that received tamsulosin alone had the lowest level of P-Smad2/3 staining, as shown in Fig. 7C.

## Discussion

The recorded mortality rate from MI has been increasing, with MI becoming the most prevalent cause of death globally. Numerous studies have demonstrated that the development of cardiac hypertrophy results from long-term activation of α1A adrenoceptors [28]. Activation of the α1A adrenoceptor subtype has been shown to cause phenylephrine-induced hypertrophy of ventricular myocytes in neonatal rats [29]. Previous studies have reported that chronic norepinephrine exposure specifically increased the level of mRNA encoding the α1A adrenoceptor in rat ventricular myocytes, which also contributed to cardiac hypertrophy and damage [30]. One of the postulated mechanisms of the detrimental effect of α1A adrenoceptor activation on the heart is that this activation is accompanied by accelerated hydrolysis of phosphoinositide and/or opening of the L-type Ca^2+^ channel, which may trigger cardiac hypertrophy, ischemia, and cardiac tissue damage [31]. In general, the effects of α1A adrenoceptors on vascular tissues, including how they mediate vasoconstriction, are well understood. However, the cardioprotective effect of α1A adrenoceptor blockade against MI has not yet been elucidated.

Tamsulosin is a strong selective antagonist of α1A adrenoceptors. Owing to its high affinity for α1A adrenoceptors, it blocks the α1A adrenoceptor-mediated positive inotropic effect [19]. It has few effects on cardiovascular extension and is well tolerated by patients with cardiovascular problems and comorbidities [32]. It is frequently prescribed for benign prostatic hyperplasia, but little is known about its capacity to protect the heart. Considerable evidence highlights its protective effects on the heart, but additional investigation and further confirmation are required.

In this study, we confirmed the ability of tamsulosin to mitigate cardiac damage caused by ISO-induced histopathological changes in cardiac tissues in rats. Considering that ISO is a synthetic catecholamine and β-adrenergic agonist, it was used in this study to induce MI in rats, which is a reliable noninvasive technique frequently used in the study of cardioprotective agents [33, 34].

Our results demonstrate that high-dose ISO induced cardiac injury and histological changes in myocardial tissues. This was proven by the increased heart weight-to-body weight ratio, which has been regarded as an index of hypertrophy. In addition, an elevated serum level of the CK-MB enzyme, as a biomarker of cardiac injury reflecting the extent of cell membrane damage and integrity, was measured.

The present data showed that long-term ISO injection in rats resulted in rapid cardiac hypertrophy and substantial cardiac injury, consistent with the findings of many previous studies that indicated that chronic ISO injection caused myocardial necrosis and fibrosis associated with cardiac injury [35–38]. Paulino et al. (2019) observed that histological analysis of sections from rat heart tissues by H&E staining revealed that the rats in the ISO-only control group displayed severe inflammatory cell infiltration, extensive edema, necrosis, and greatly dilated and engorged blood vessels compared with the rats in the control group [39].

According to research findings reported in the literature, administration of high doses of ISO can result in strong cardiac activity, an imbalanced oxygen supply, increases in the production of free radical species and MI biomarkers, myocardial necrosis, and cardiac remodeling in response to the adaptive mechanisms of the heart [34]. These explanations may support our findings that tamsulosin treatment significantly reduced the heart weight-to-body weight ratio and CK-MB level in rats treated with ISO. In addition, a previous study revealed that oxidative stress is increased in myocardial tissues in response to MI [40–42]. When compared with the control rats, the rats that received ISO injections showed significantly increased ROS production and oxidative stress, which caused MI. Conversely, α1A adrenoceptor inhibition reduced the amount of oxidative stress that could lead to myocardial injury [43, 44]. Although tamsulosin administration decreased the levels of oxidative stress biomarkers, to the best of our knowledge, no supporting evidence that confirms the effects of α1A adrenoceptor inhibitors on oxidative stress biomarkers, especially in cardiac tissues, has been reported in the literature. The activation of fibroblasts that remodel the myocardium and promote matrix preservation is accomplished by inducing the activity of TGF-β, a key mediator of fibrogenesis, through pathways involving intracellular molecules such as Smads or Smad-independent cascades [45].

The ability of ISO to cause myocardial fibrosis in rats was demonstrated by the increased TGF-β, collagen III, and phosphorylated Smad2/Smad3 protein levels. Matrix metalloproteinases (MMPs) are selective enzymes involved in the remodeling of the cardiac extracellular matrix (ECM) [46]. An earlier investigation revealed that proinflammatory cytokines and MMPs were more highly expressed during ISO-mediated fibrosis [47]. Numerous studies support our findings, showing that ISO induces cardiac fibrosis and plays a role in the upregulation of TGF-β/Smad2/Smad3 expression in cardiac tissues in a rat model of ISO-induced MI [48]. Another study that provided strong evidence that ISO causes cardiac fibrosis showed abnormally arranged myocardial tissue that was heavily populated with disordered collagen fibers, which resulted in fibrotic cardiac remodeling [49]. All of these studies, including our own, showed a connection between ISO administration and ensuing cardiac hypertrophy, MI, myocyte necrosis, fibroblast proliferation, and connective tissue accumulation [50]. In vitro exposure of cardiac fibroblasts to superoxide anions stimulates their proliferation by increasing the production of TGF-β, a potent fibrogenic cytokine. This finding supports the link between the increased oxidative stress during cardiac injury, the induction of TGF-β activity and the ultimately mediated cardiac fibrosis [51].

Through the α1A adrenoceptor/p38/Smad3 signaling pathway, sympathetic overactivity promotes epithelial–mesenchymal transition (EMT) in renal epithelial cells and fibrosis, whereas α1A adrenoceptor inhibition may receive attention in the future owing to its potential effectiveness for the treatment of renal fibrosis [52]. In the present study, tamsulosin significantly decreased ISO-induced cardiac fibrosis, possibly as a result of its free radical scavenging and antioxidant activities, which may be responsible for inhibiting collagen synthesis and preventing the ISO-induced accumulation of collagen. The observed antifibrotic effects of tamsulosin are consistent with a previous study indicating that TGF-β/CTGF signaling is suppressed by the adrenergic receptor inhibitor carvedilol, which improves biventricular fibrosis and function [53]. This study further elucidates that by lowering the protein levels of collagen III, TGF-β, and phosphorylated Smad2/Smad3, tamsulosin mediates antifibrotic effects on infarcted cardiac tissues.

ILK is a member of the serine/threonine protein kinase family and plays a critical role in the transmission of biomechanical signals derived from cell–matrix interactions. By binding to the cytoplasmic domain of β-integrins, ILK controls cytoskeletal remodeling and is linked to cardiac contractility, ventricular hypertrophy, and cardiac repair [54]. Evidence from a previous study indicated that targeted ILK deletion caused dilated cardiomyopathy and spontaneous heart failure in murine hearts, supplementing the knowledge about the function of ILK in heart failure [55].

Fibrosis may be related to increased integrin expression in specific cell types. In addition to the direct effects of ILK on cellular proliferation, migration, and survival, integrins act as receptors for matricellular proteins and potentiate signals from soluble growth factors such as TGF-β. These effects are caused by the binding of integrins to ECM proteins. This suggests that integrins and the proteins that interact with them are crucial to the development of fibrosis. The fibrotic process clearly benefits from increased integrin expression [56, 57]. Previous studies have shown that animals given ILK showed improvements in cardiac function, including decreases in the infarct size, interstitial fibrosis, and apoptosis [54, 58].

According to these studies, ILK overexpression may protect cardiac function and lessen the risk of post-MI cardiac fibrosis, whereas downregulation of ILK expression may contribute to cardiac injury. The results of the present study suggest a critical role of tamsulosin administration in reversing the attenuation of ILK expression after ISO-induced MI that is associated with increased TGF-β1 expression. Accumulating evidence on kidney diseases suggests that ILK ablation in vivo diminishes the expression of the EMT marker α-smooth muscle actin (α-SMA) and the inducer TGF-β1 in renal tubular epithelial cells in a model of cisplatin-induced acute kidney injury [59–61].

**Fig. 8:**
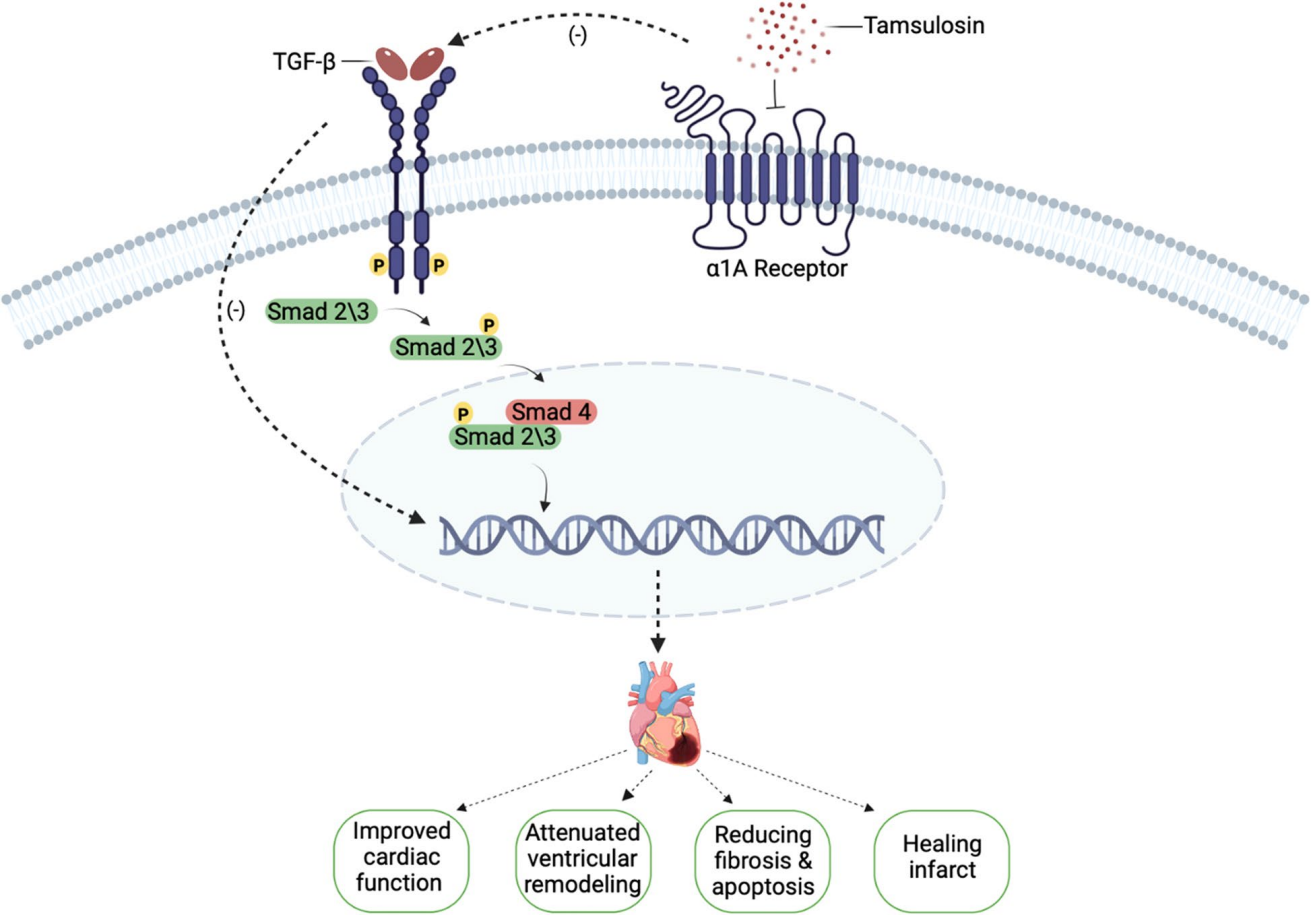
Graphical abstract showing the specific theme and aims of this study

The association between the effects of tamsulosin treatment on the infarcted heart in rat model of ISO-induced MI and the protein levels of ILK/TGF-/p-Smad2/Smad3 has not yet been established, even though the multifunctional effector ILK is linked to integrin receptors and has been demonstrated to actively promote cardiac hypertrophy while interacting synergistically with other hypertrophic stimuli such as the α-adrenergic agonist phenylephrine. In light of this finding, the present study is important in that it elucidates this relationship and confirms the cardioprotective effect of tamsulosin in MI (Fig. 8).

This study has a few limitations, but these might not be important enough to impact the results. One limitation is related to the potential ethical issue of using a limited number of animal samples (only six rats per group), which might be inadequate and provide little scientific and statistical power. However, a considerable number of published research studies have used this sample size. In addition, although in vivo hemodynamic parameter assessments provide valuable information on cardiovascular effects, in this study, we relied on determining the heart weight-to-body weight ratio as an index of cardiac hypertrophy and on measuring the levels of cardiac injury biomarkers such as troponin I and CK-MB, in addition to the assessment of heart tissue damage using histological examination.

In conclusion, our findings indicate that tamsulosin, an α1A adrenoceptor blocker, plays a key role in exerting a cardioprotective effect by inhibiting the binding of inflammatory, oxidative, and fibrosis-related mediators. In addition, this study suggests that α1A adrenoceptor inhibition may be a promising new therapeutic strategy for MI. Furthermore, this study sheds light on the roles of the ILK and TGF-β/SMAD2/3 signaling pathways in the development of MI and how these signaling pathways could be modulated by tamsulosin and hence contribute to its cardioprotective effect in the setting of MI. However, a clinical study should be considered to evaluate the efficacy, safety, and tolerability of α1A adrenoceptor blockers as a treatment option for MI.

## Supplementary Information

**Additional file 1.**

## Data Availability

The datasets used and analyzed and the raw data supporting all tables and figures have been provided by the authors in PDF format in the Supplementary Information file separate from the original manuscript.

## References

[1] Heart-Health Screenings. www.Heart.org. 2021. Available from: https://www.heart.org/en/health-topics/consumer-healthcare/what-is-cardiovascular-disease/heart-health-screenings.

[2] Moradi-Arzeloo M, Farshid AA, Tamaddonfard E, Asri-Rezaei S (2016). Effects of histidine and vitamin C on isoproterenol-induced acute myocardial infarction in rats. Vet Res Forum.

[3] Wang H, Eitzman D (2013). Acute myocardial infarction leads to acceleration of atherosclerosis. Atherosclerosis.

[4] Hung MJ, Hu P, Hung MY (2014). Coronary artery spasm: Review and update. Int J Med Sci.

[5] Zhao X, Balaji A, Pachon R, Beniamen D, Vatner D, Graham R (2015). Overexpression of cardiomyocyte α1A-adrenergic receptors attenuates postinfarct remodeling by inducing angiogenesis through heterocellular signaling. Arterioscler Thromb Vasc Biol.

[6] Lu W, Xie J, Gu R, Xu B (2017). Expression of integrin-linked kinase improves cardiac function in a swine model of myocardial infarction. Exp Ther Med.

[7] Wu Y, Yin X, Wijaya C, Huang MH, McConnell BK (2011). Acute myocardial infarction in rats. J Vis Exp.

[8] Oka T, Akazawa H, Naito A, Komuro I (2014). Angiogenesis and Cardiac Hypertrophy. Circ Res.

[9] Talman V, Ruskoaho H (2016). Cardiac fibrosis in myocardial infarction-from repair and remodeling to regeneration. Cell Tissue Res.

[10] Shinde A, Frangogiannis N (2014). Fibroblasts in myocardial infarction: a role in inflammation and repair. J Mol Cell Cardiol.

[11] Mahabeleshwar G, Feng W, Phillips D, Byzova T (2006). Integrin signaling is critical for pathological angiogenesis. J Exp Med.

[12] Giancotti F, Ruoslahti E (1999). Integrin signaling. Science.

[13] Goumans MJ, Lebrin F, Valdimarsdottir G (2003). Controlling the angiogenic switch: a balance between two distinct TGF-β receptor signaling pathways. Trends Cardiovasc Med.

[14] Bujak M, Frangogiannis NG (2006). The role of TGF-beta signaling in myocardial infarction and cardiac remodeling. Cardiovasc Res.

[15] Yu CM, Tipoe GL, Wing-Hon Lai K, Lau CP (2001). Effects of combination of angiotensin-converting enzyme inhibitor and angiotensin receptor antagonist on inflammatory cellular infiltration and myocardial interstitial fibrosis after acute myocardial infarction. J Am Coll Cardiol.

[16] Nurgazieva D, Mickley A, Moganti K, Ming W, Ovsyi I, Popova A (2015). TGF beta1, but not bone morphogenetic proteins, activates Smad1/5 pathway in primary human macrophages and induces expression of proatherogenic genes. J Immunol.

[17] Shiojima I, Walsh K (2002). Role of Akt signaling in vascular homeostasis and angiogenesis. Circ Res.

[18] Gu R, Bai J, Ling L, Ding L, Zhang N, Ye J (2012). Increased expression of integrin-linked kinase improves cardiac function and decreases mortality in dilated cardiomyopathy model of rats. PLoS ONE.

[19] Jensen BC (2014). OʼConnell TD, Simpson PC: Alpha-1-adrenergic receptors in heart failure: the adaptive arm of the cardiac response to chronic catecholamine stimulation. J Cardiovasc Pharmacol.

[20] Marks LS, Gittelman MC, Hill LA, Volinn W, Hoel G (2009). Rapid efficacy of the highly selective alpha-1A-adrenoceptor antagonist silodosin in men with signs and symptoms of benign prostatic hyperplasia: pooled results of 2 phase 3. J Urol.

[21] Yu HJ, Lin A, Yang S, Tsui KH, Wu HC, Cheng CL (2011). Non-inferiority of silodosin to tamsulosin in treating patients with lower urinary tract symptoms (LUTS) associated with benign prostatic hyperplasia (BPH). BJU Int.

[22] Miyazawa Y, Paul Starkey L, Forrest A, Schentag JJ, Kamimura H, Swarz H, Ito Y (2002). Effects of the concomitant administration of tamsulosin (0.8 mg) on the pharmacokinetic and safety profile of intravenous digoxin (Lanoxin) in normal healthy subjects: a placebo-controlled evaluation. J Clin Pharm Ther..

[23] Pingree SD, Simmonds PL, Woods JS (2001). Effects of 2,3-dimercapto-1-propanesulfonic Acid (DMPS) on tissue and urine mercury levels following prolonged methylmercury exposure in rats. Toxicol Sci.

[24] Moron MS, Depierre J, Mannervik B (1979). Levels of glutathione, glutathione reductase and glutathione S-transferase activities in rat lung and liver. Biochem Biophys Acta.

[25] Ohkawa H, Ohishi N, Yagi K (1979). Assay for lipid peroxides in animal tissues by thiobarbituric acid reaction. Anal Biochem.

[26] Delides A, Spooner RJ, Goldberg DM, Neal FE (1976). An optimized semi-automatic rate method for serum glutathione reductase activity and its application to patients with malignant disease. J Clin Pathol.

[27] Towbin H, Staehelin T, Gordon J (1979). Electrophoretic transfer of proteins from polyacrylamide gels to nitrocellulose sheets: procedures and some applications. Proc Natl Sci USA.

[28] Hieble JP (2000). Adrenoceptor subclassification: an approach to improved cardiovascular therapeutics. Pharm Acta Helv.

[29] Garcia-Sainz JA, Alcantara-Hernandez R, Vazquez-Prado J (1998). α1-Adrenoceptor subtype activation increases proto-oncogene mRNA levels. Role of protein kinase C. Eur J Pharmacol..

[30] Rokosh DG, Stewart AF, Chang KC, Bailey BA, Karliner JS, Camacho SA (1996). α1-Adrenergic receptor subtype mRNAs are differentially regulated by α1-adrenergic and other hypertrophic stimuli in cardiac myocytes in culture and *in vivo*. J Biol Chem.

[31] Yang HT, Endoh M (1996). (±)-Tamsulosin, an α1A-adrenoceptor antagonist, inhibits the positive inotropic effect but not the accumulation of inositol phosphates in rabbit heart. Eur J Pharmacol.

[32] Honda KT, Takenaka A, Miyata-Osawa M, Terai K, Shiono T (1985). Studies on YM-12617: a selective and potent antagonist of postsynaptic α1-adrenoceptors. Naunyn Schmiedebergs Arch Pharmacol..

[33] Chapple CR (2005). A comparison of varying alpha-blockers and other pharmacotherapy options for lower urinary tract symptoms. Rev Urol.

[34] Brooks WW, Conrad CH (2009). Isoproterenol-induced myocardial injury and diastolic dysfunction in mice structure and functional correlate. Comp Med.

[35] Upaganlawar A, Gandhi H, Balaraman R (2011). Isoproterenol induced myocardial infarction: protective role of natural products. J Pharmacol Toxicol.

[36] Guo B, Li YJ, Han R, Yang SL, Shi YH, Han DR (2011). Telmisartan attenuates isoproterenol-induced cardiac remodeling in rats via regulation of cardiac adiponectin expression. Acta Pharmacol Sin.

[37] Kondo T, Ogawa Y, Sugiyama S, Ito T, Satake T, Ozawa T (1987). Mechanism of isoproterenol induced myocardial damage. Cardiovasc Res.

[38] Kudej RK, Iwase M, Uechi M, Vatner DE, Oka N, Ishikawa Y (1997). Effects of chronic beta-adrenergic receptor stimulation in mice. J Mol Cell Cardiol.

[39] Paulino ET, Ferreira AKB, Da Silva JCG, Costa CDF, Smaniotto S, De Araűjo-Jűnior JX (2019). Cardioprotective effects induced by hydroalcoholic extract of leaves of *alpinia zerumbet* on myocardial infarction in rats. J Ethnopharmacol.

[40] Kurian G, Rajagopal R, Vedantham S, Rajesh M (2016). The Role of oxidative stress in myocardial ischemia and reperfusion injury and remodeling: revisited. Oxid Med Cell Longev.

[41] Davel A, Brum P, Rossoni L (2014). Isoproterenol induces vascular oxidative stress and endothelial dysfunction via a Giα-coupled β2-adrenoceptor signaling pathway. PLoS ONE.

[42] Singal PK, Khaper N, Palace V, Kumar D (1998). The role of oxidative stress in the genesis of heart disease. Cardiovasc Res.

[43] Chen H, Xu Y, Wang J, Zhao W, Ruan H (2015). Baicalin ameliorates isoproterenol-induced acute myocardial infarction through iNOS, inflammation and oxidative stress in rat. Int J Clin Exp Pathol.

[44] Fan Y (2019). Cardioprotective effect of rhapontigenin in isoproterenol-induced myocardial infarction in a rat model. Pharmacology.

[45] Frangogiannis NG (2019). Cardiac fibrosis: Cell biological mechanisms, molecular pathways and therapeutic opportunities. Mol Aspects Med.

[46] Gao HC, Zhao H, Zhang WQ, Li YQ (2013). The role of the Rho/Rock signaling pathway in the pathogenesis of acute ischemic myocardial fibrosis in rat models. Exp Ther Med.

[47] Feng W, Li W (2010). The study of ISO induced heart failure rat model. Exp Mol Pathol.

[48] Wang Q, Yu X, Xu H, Zhao X, Sui D (2019). Ginsenoside re improves isoproterenol-induced myocardial fibrosis and heart failure in rats. Evid Based Complement Alternat Med.

[49] Wu X, Li M, Chen SQ, Li S, Guo F (2018). Pin1 facilitates isoproterenol-induced cardiac fibrosis and collagen deposition by promoting oxidative stress and activating the MEK1/2-ERK1/2 signal transduction pathway in rats. Intl J Mol Med.

[50] Benjamin I, Jalil J, Tan L, Cho K, Weber K, Clark W (1989). Isoproterenol-induced myocardial fibrosis in relation to myocyte necrosis. Circ Res.

[51] Purnomo Y, Piccart Y, Coenen T, Prihadi J, Lijnen P (2013). Oxidative stress and transforming growth factor-β-induced cardiac fibrosis. Cardiovasc Hematol Disord Drug Targets.

[52] Ren H, Zuo S, Hou Y, Shang W, Liu N, Yin Z (2020). Inhibition of α1-adrenoceptor reduces TGF-β1-induced epithelial-to-mesenchymal transition and attenuates UUO-induced renal fibrosis in mice. FASEB J.

[53] Okumura K, Kato H, Honjo O, Breitling S, Kuebler WM, Sun M (2015). Carvedilol improves biventricular fibrosis and function in experimental pulmonary hypertension. J Mol Med (Berl).

[54] Hannigan GE, Coles JG, Dedhar S (2007). Inegrin-linked kinase at heart of cardiac contractility, repair, and disease. Cir Res.

[55] White DE, Coutu P, Shi YF, Tardif JC, Nattel S, Arnaud RS (2006). Targeted ablation of ILK from the murine heart results in dilated cardiomyopathy and spontaneous heart failure. Genes Dev.

[56] Chen C, Li R, Ross RS, Manso AM (2016). Integrins and integrin-related proteins in cardiac fibrosis. J Mol Cell Cardiol.

[57] Sofia RR, Serra AJ, Silva JA, Antonio EL, Manchini L (2014). Gender-based differences in cardiac remodeling and ILK expression after myocardial infarction. Arq Bras Cardiol.

[58] Ding L, Dong L, Chen X, Zhang L, Xu X, Ferro A (2009). Increased expression of integrin-linked kinase attenuation left ventricular remodeling and improves cardiac function after myocardial infarction. Circulation.

[59] Chen H, Huang XN, Yan W, Chen K, Guo L, Tummalapali L (2005). Role of the integrin-linked kinase/PINCH1/alpha-parvin complex in cardiac myocyte hypertrophy. Lab Invest.

[60] Lu H, Fedak PW, Dai X, Du C, Zhou YQ, Henkelman M (2006). Integrin-linked kinase expression Is elevated in human cardiac hypertrophy and induces hypertrophy in transgenic mice. Circulation.

[61] Cano-Peńalver JL, Griera M, Garcȋa-Jerez M, Hatem-Vaquero M, Ruiz-Torres MP, Rodrìguez-Puyol D (2016). Renal integrin-linked kinase depletion induces kidney cGMP axis upregulation: consequences on basal and acutely damaged renal function. Mol Med.

