# Supplementary material for: α1A Adrenoreceptor blockade attenuates myocardial infarction by modulating the integrin-linked kinase/TGF-β/Smad signaling pathways
Source: BMC Cardiovasc Disord. 2023 Mar 24;23:153. doi: 10.1186/s12872-023-03188-w (PMC10037904; doi:10.1186/s12872-023-03188-w)
Supplement: Supplementary file 1 — Additional file 1. [file 12872_2023_3188_MOESM1_ESM.zip › Original data for Figure 4.Tamsulosin modulates the beta-actin protein expression level in cardiomyocytes after MI.pdf]

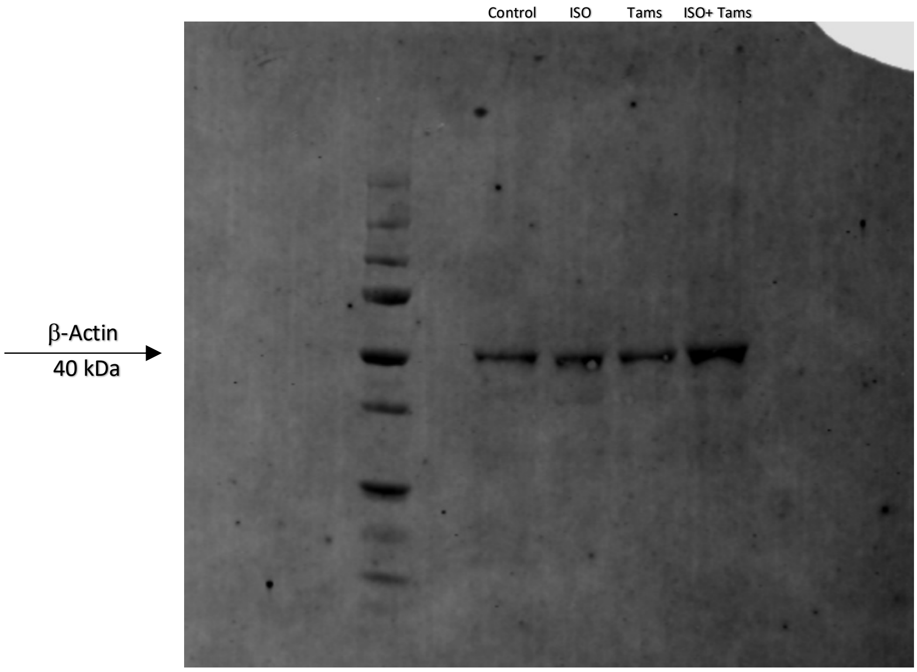

Set no. 1 control_ISO_Tam_Tam and ISO

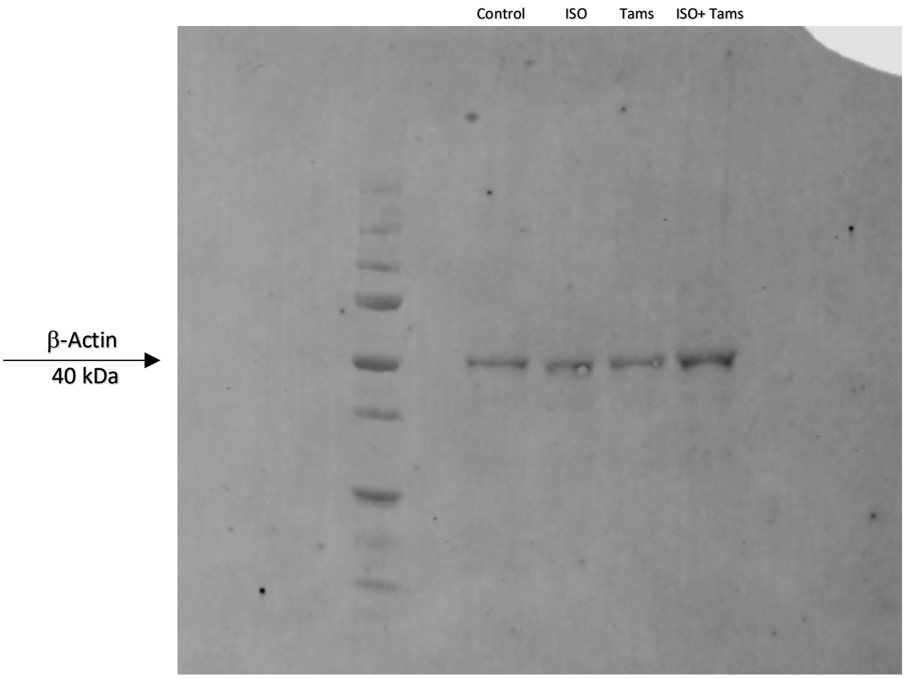

Set no. 2 β-Actin control_ISO_Tam_Tam and ISO

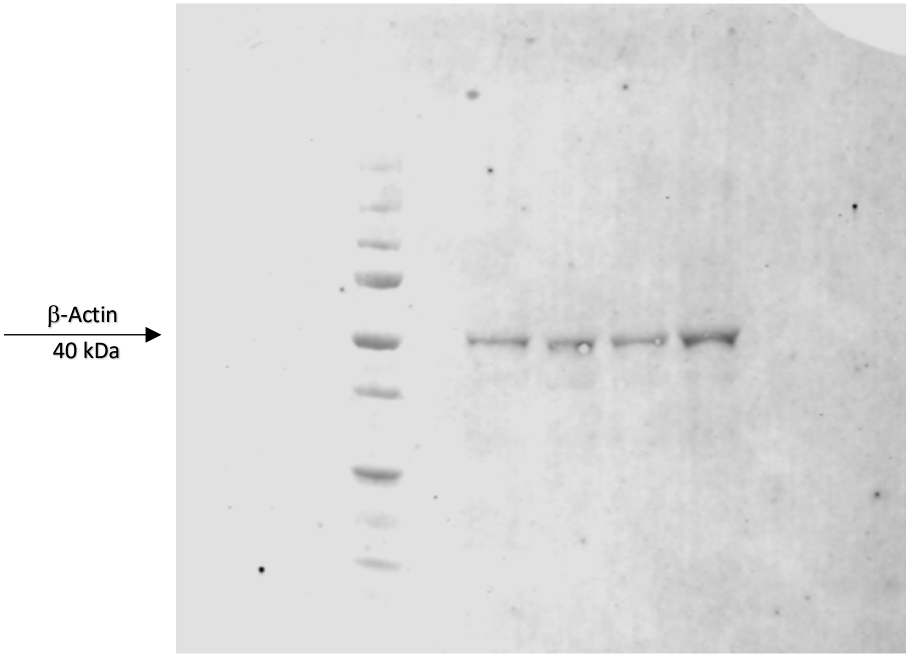

Set no. 3 β-Actin control_ISO_Tam_Tam and ISO

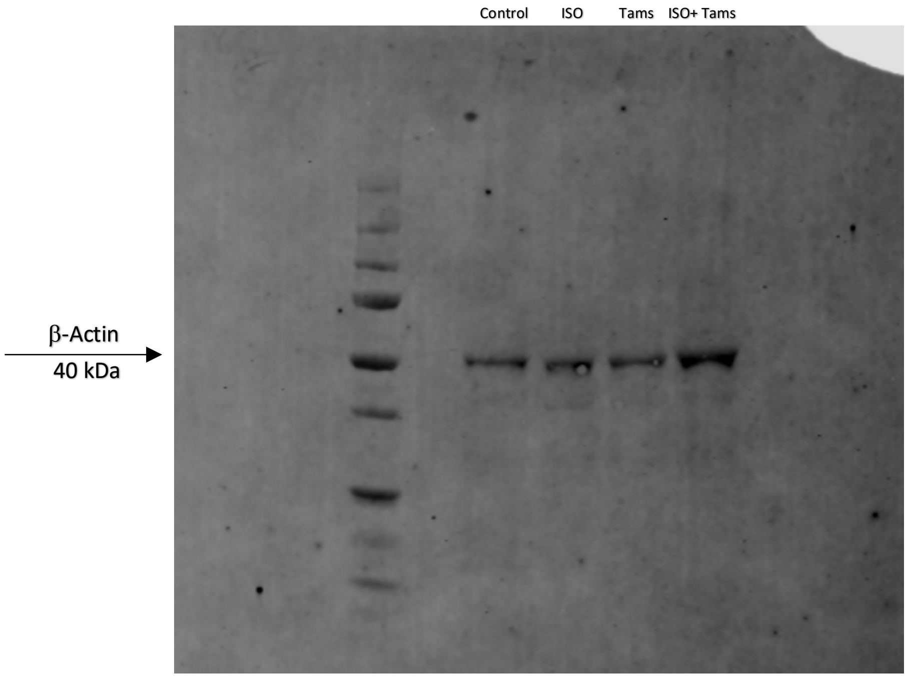

Set no. 4 β-Actin control_ISO_Tam_Tam and ISO

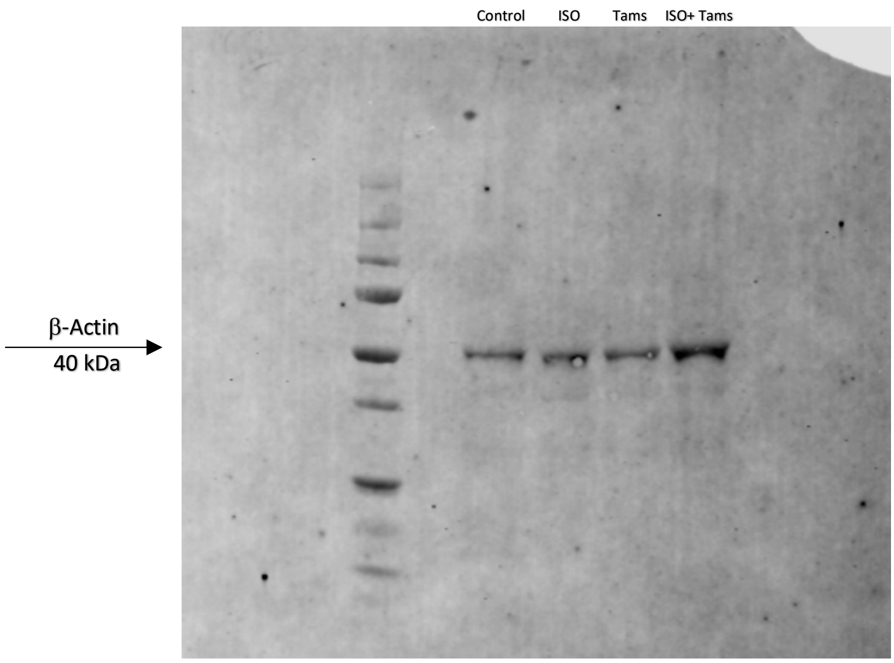

Set no. 5 β-Actin control_ISO_Tam_Tam and ISO

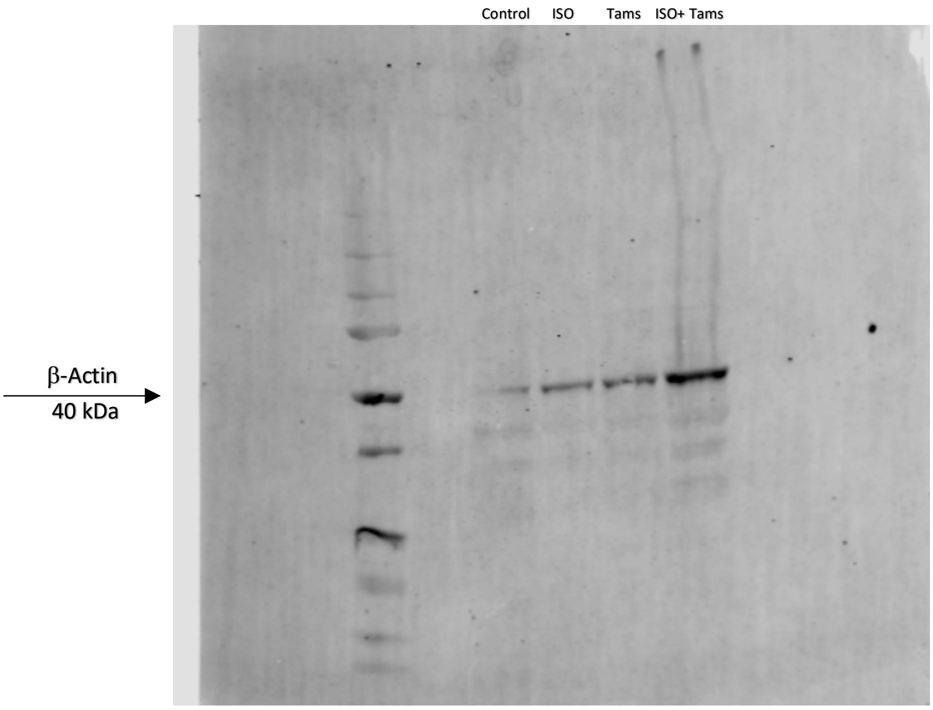

Set no. 6 β-Actin control_ISO_Tam_Tam and ISO
