# Supplementary material for: α1A Adrenoreceptor blockade attenuates myocardial infarction by modulating the integrin-linked kinase/TGF-β/Smad signaling pathways
Source: BMC Cardiovasc Disord. 2023 Mar 24;23:153. doi: 10.1186/s12872-023-03188-w (PMC10037904; doi:10.1186/s12872-023-03188-w)
Supplement: Supplementary file 1 — Additional file 1. [file 12872_2023_3188_MOESM1_ESM.zip › original data for figure no. 4. Tamsulosin modulates ILK protein expression levels in cardiomyocyte after MI. .pdf]

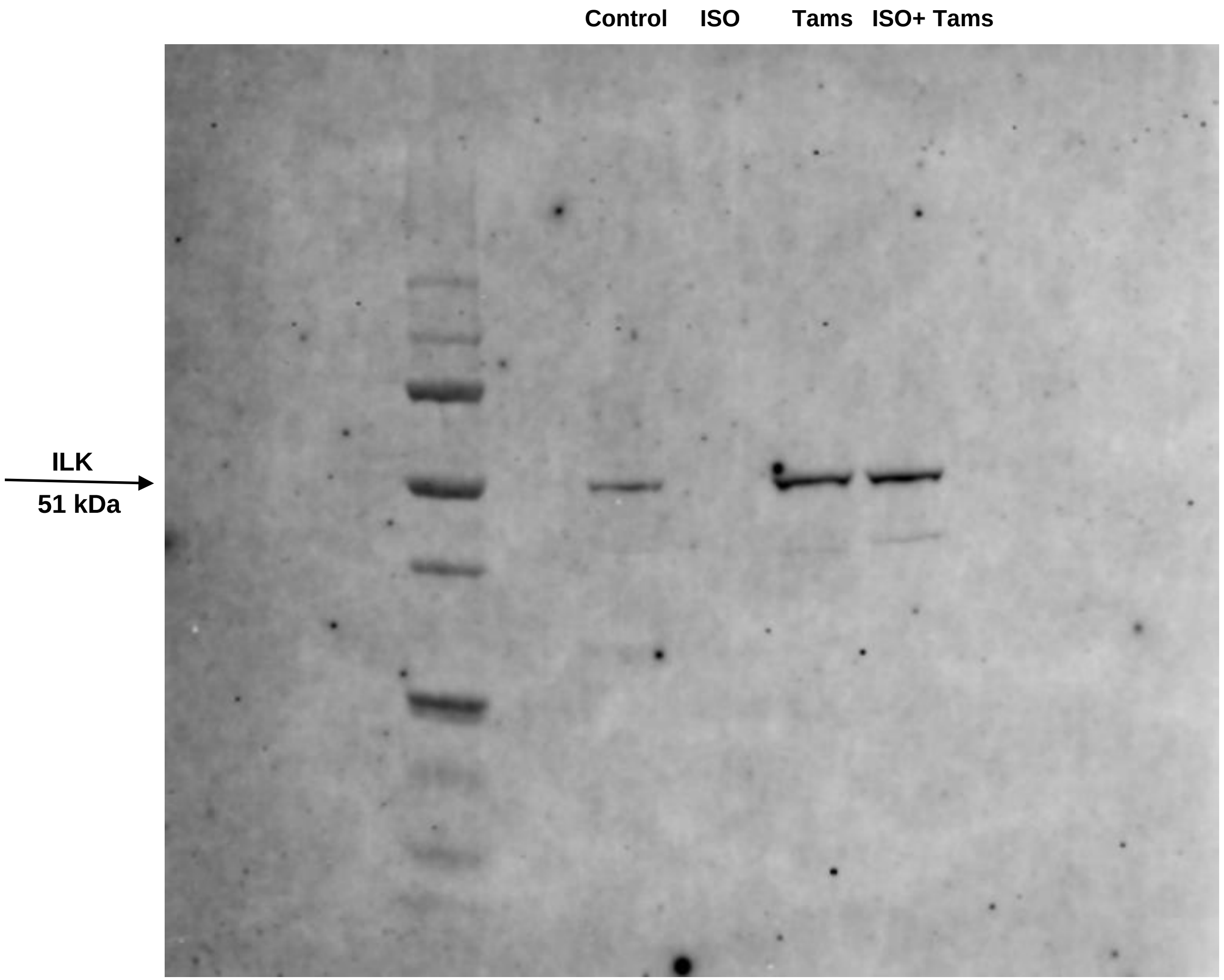

Set no.1 ILK control_ ISO_ Tam_ Tam and ISO

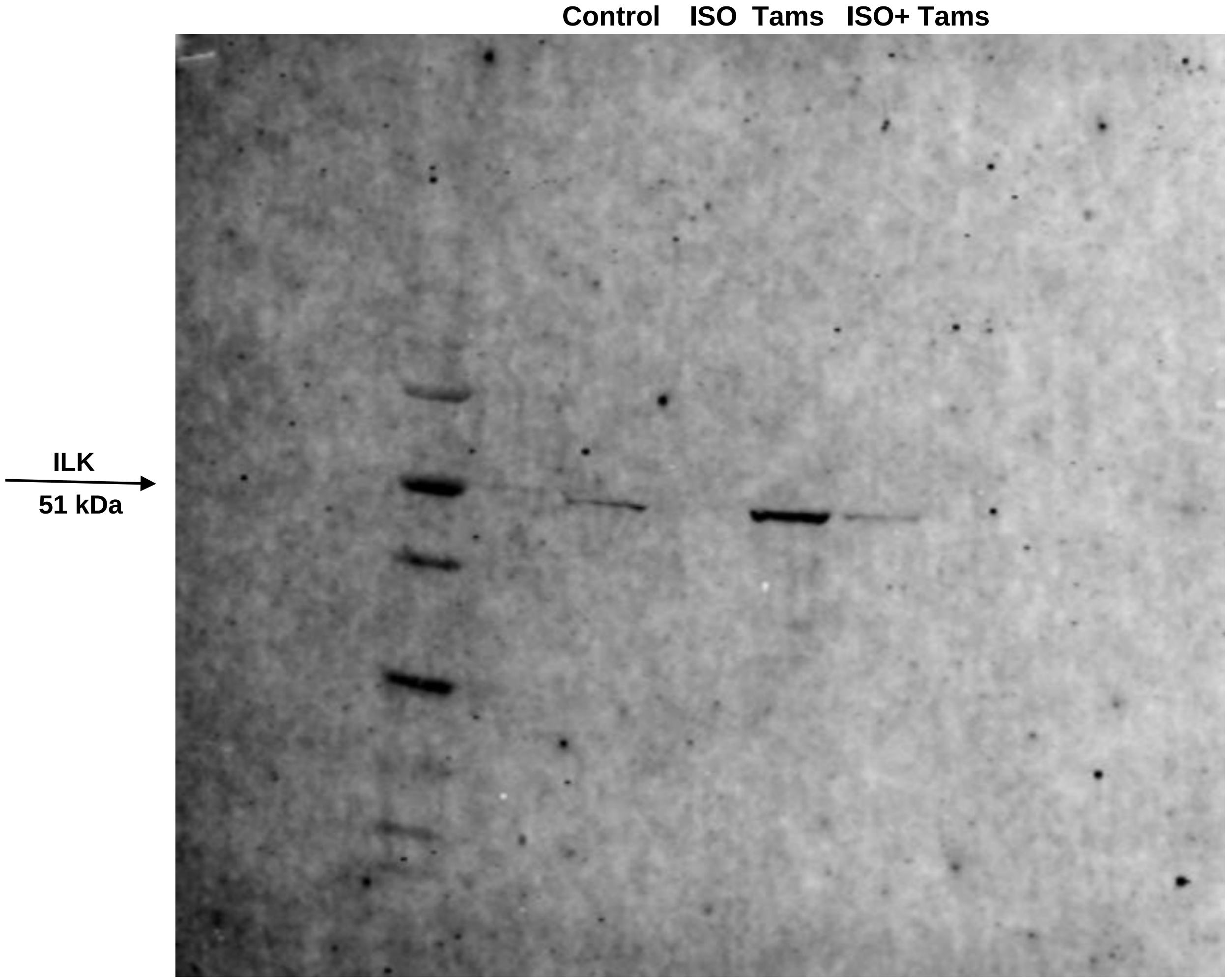

Set no.2 ILK control_ ISO_ Tam_ Tam and ISO

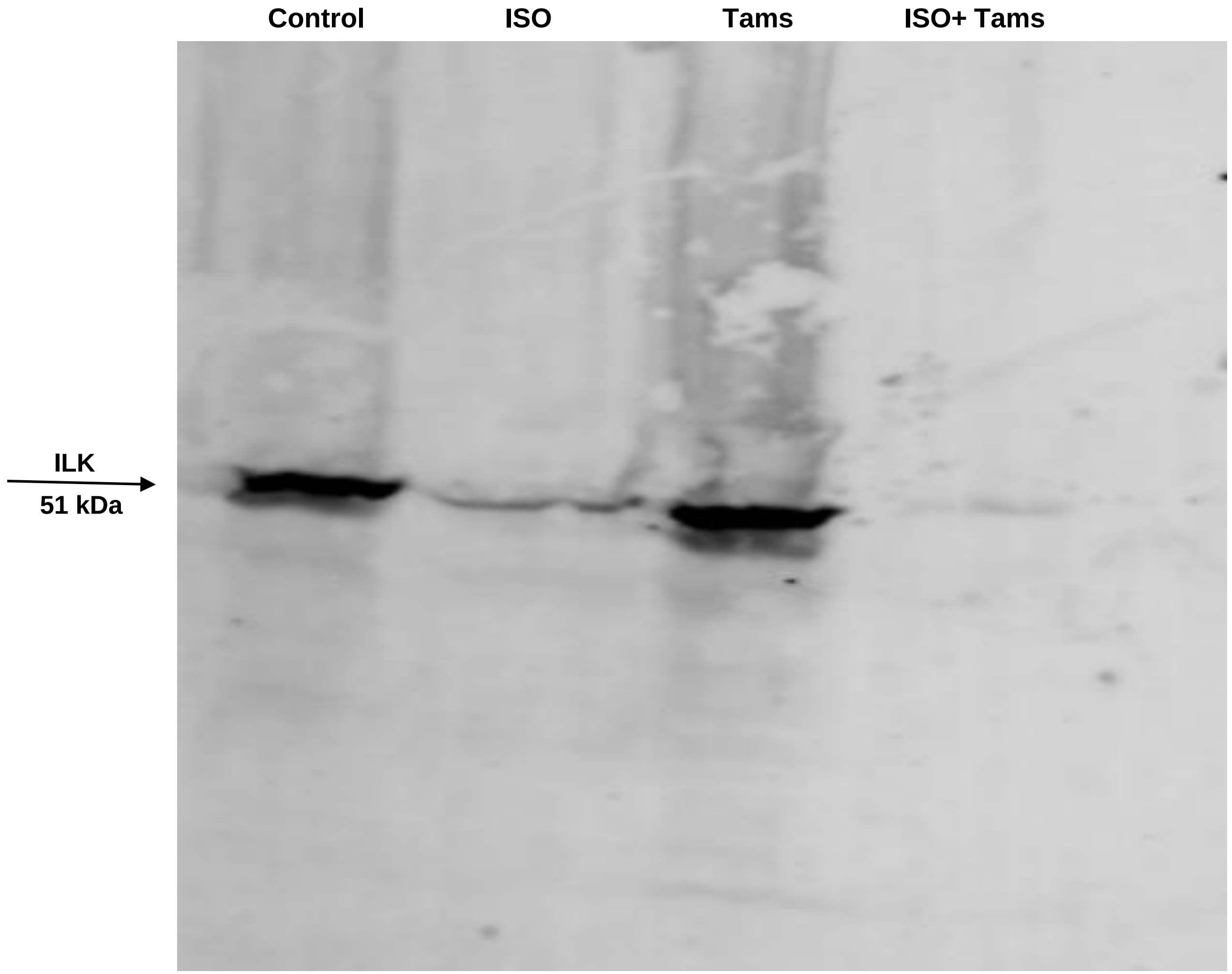

Set no.3 ILK control_ ISO_ Tam_ Tam and ISO

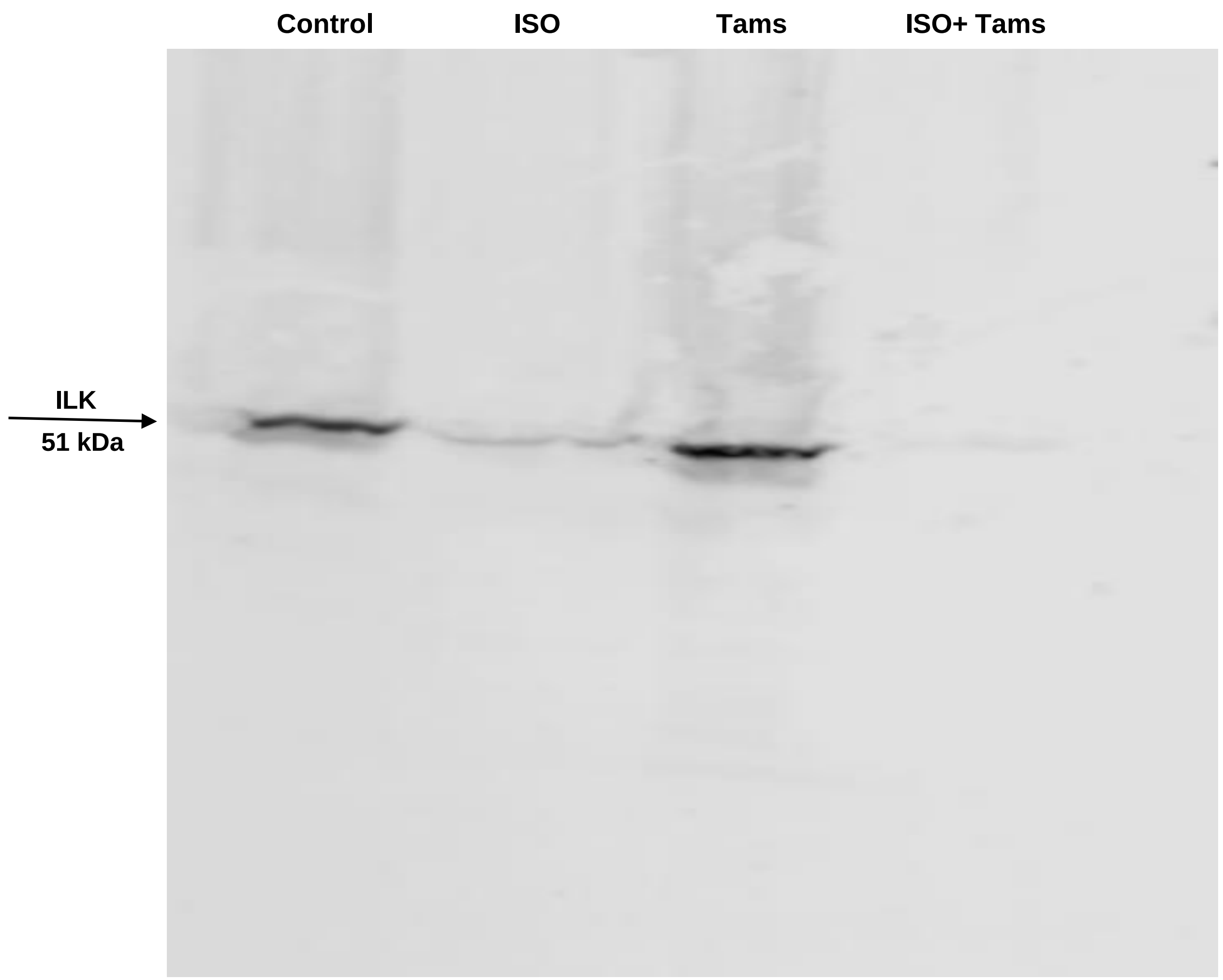

Set no.4 ILK control_ ISO_ Tam_ Tam and ISO

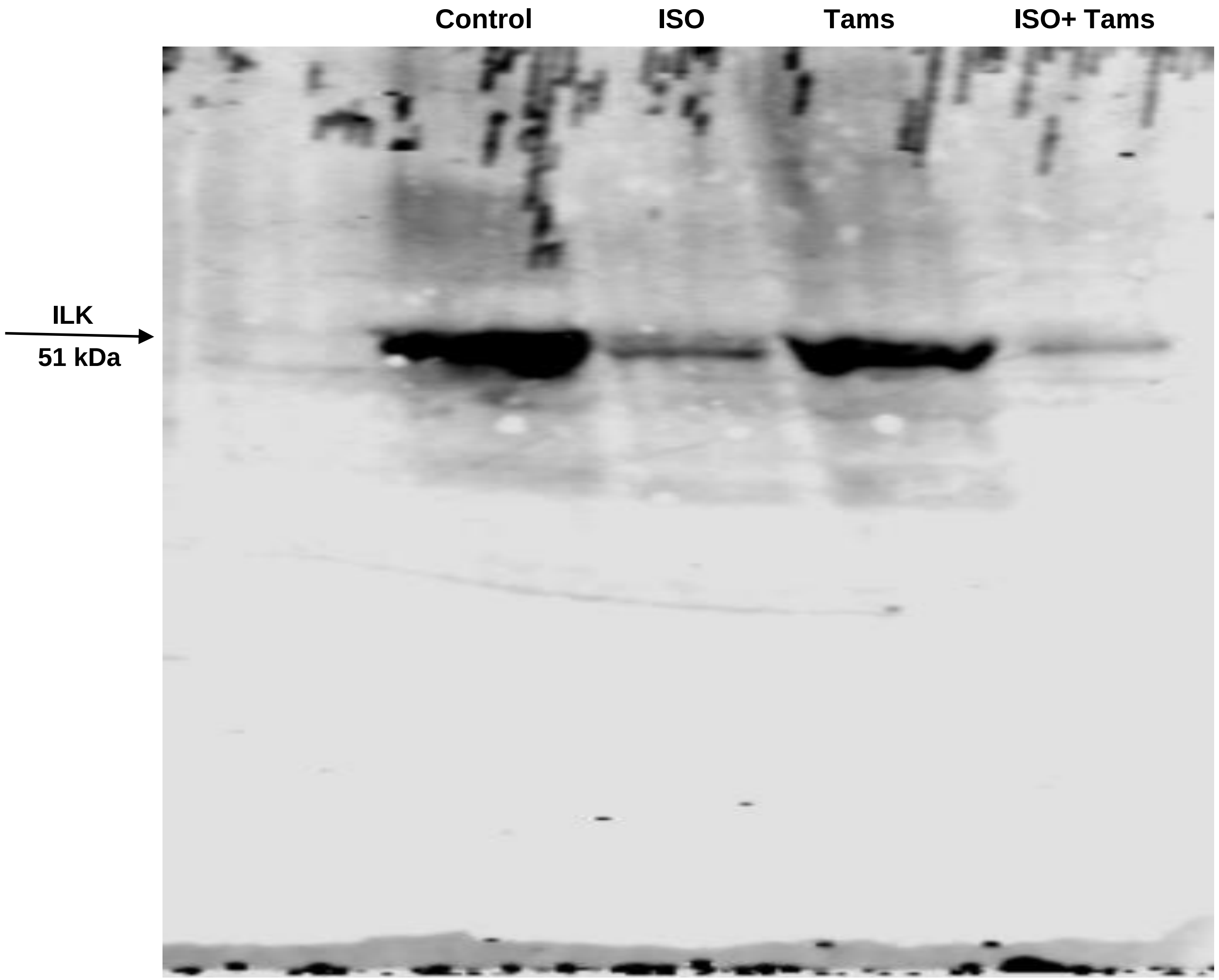

Set no.5 ILK control_ ISO_ Tam_ Tam and ISO

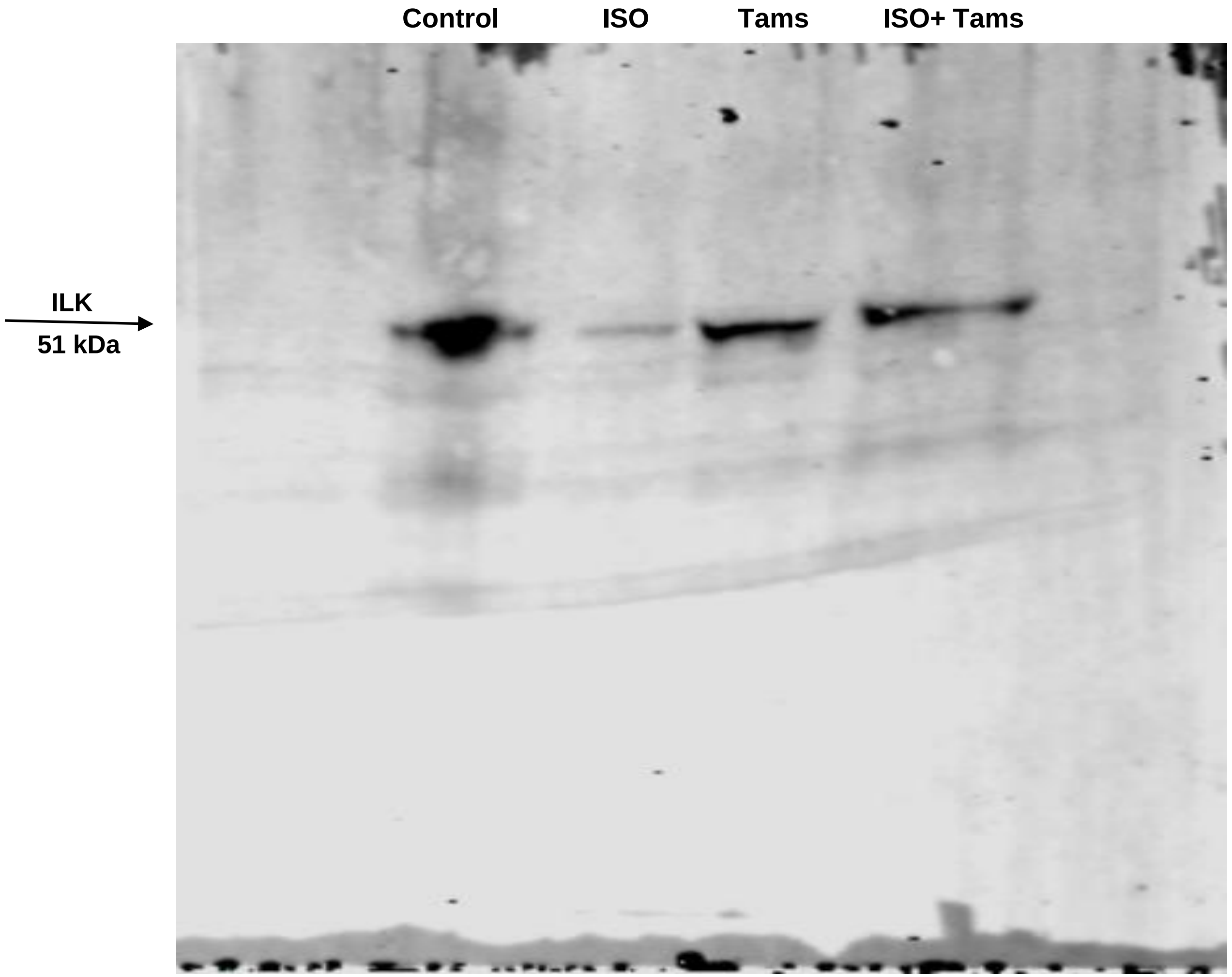

Set no.6 ILK control_ ISO_ Tam_ Tam and ISO
