# Supplementary figures and images for: α1A Adrenoreceptor blockade attenuates myocardial infarction by modulating the integrin-linked kinase/TGF-β/Smad signaling pathways
Source: BMC Cardiovasc Disord. 2023 Mar 24;23:153. doi: 10.1186/s12872-023-03188-w (PMC10037904; doi:10.1186/s12872-023-03188-w)

Control

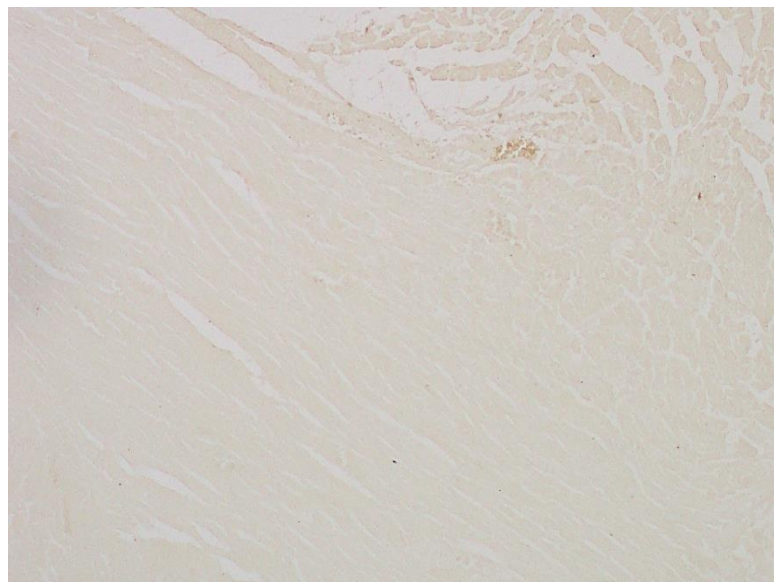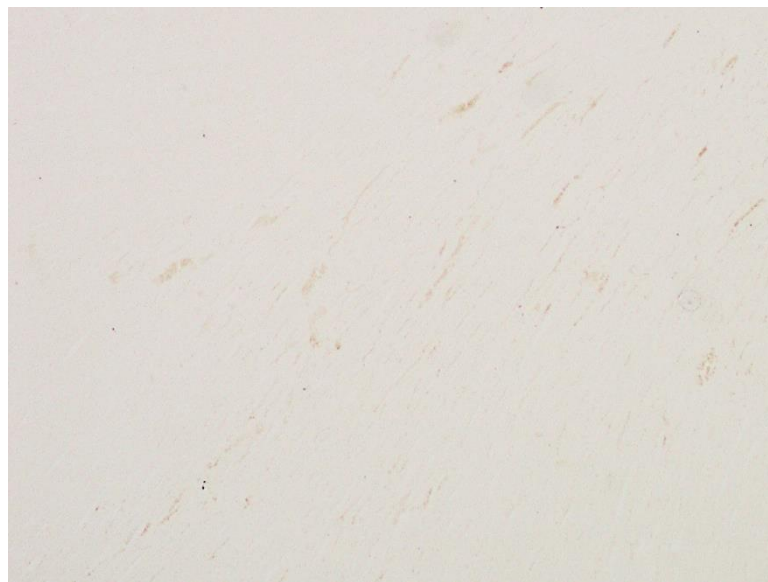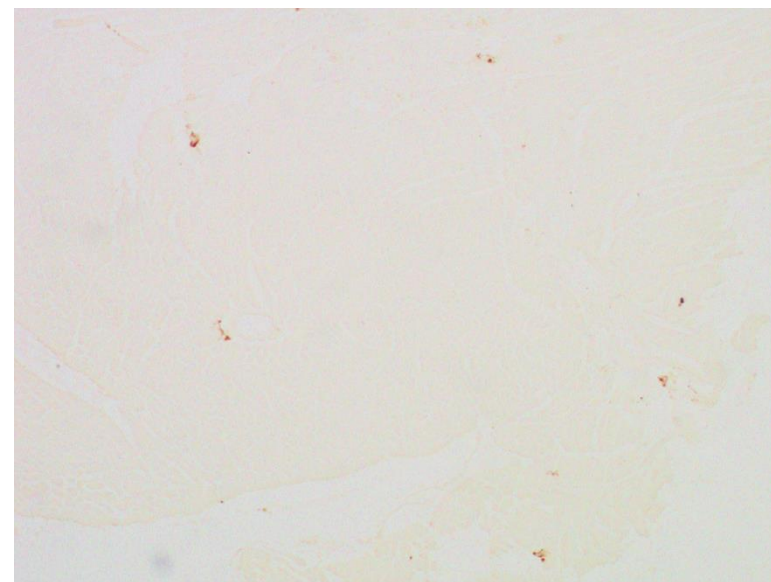

ISO

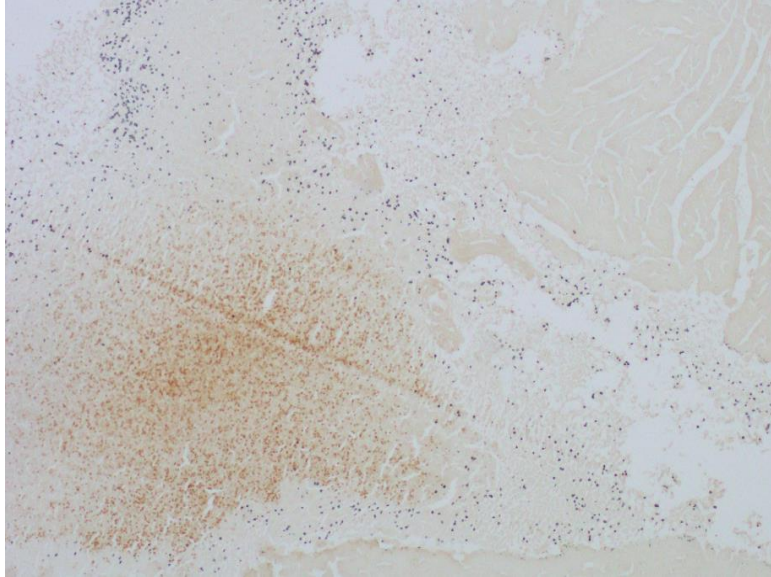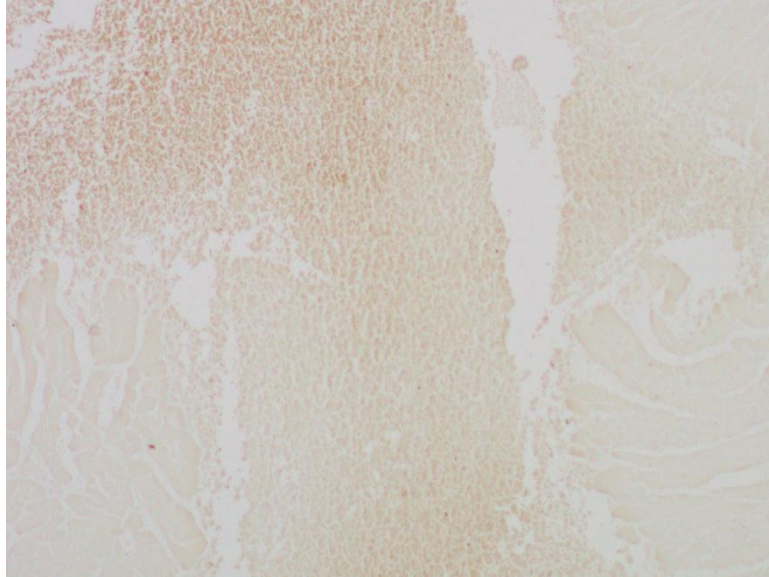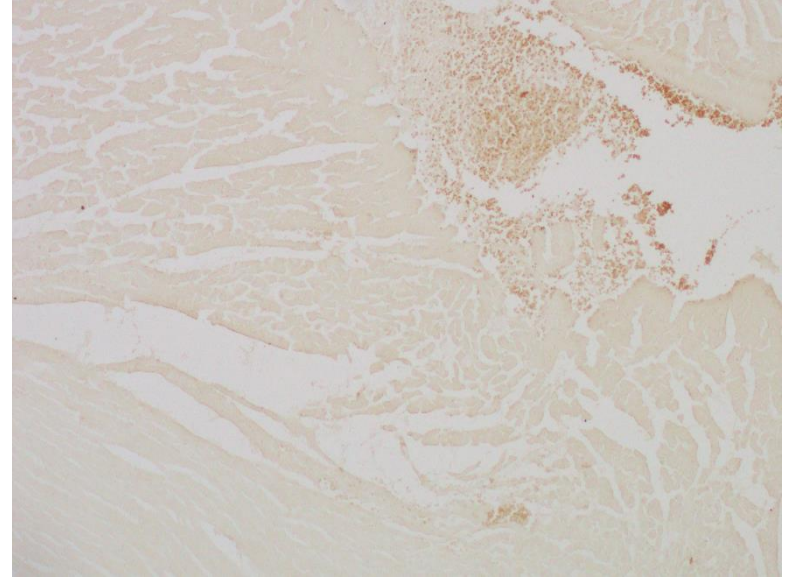

## Tamsulosin

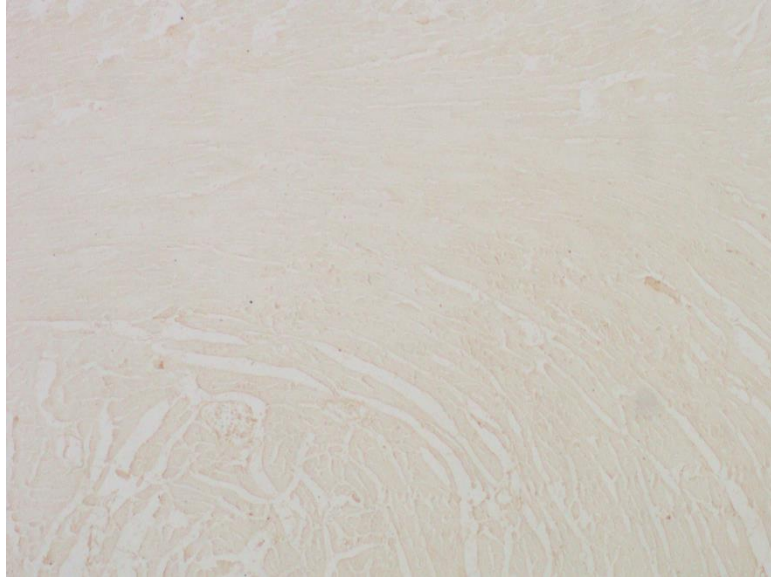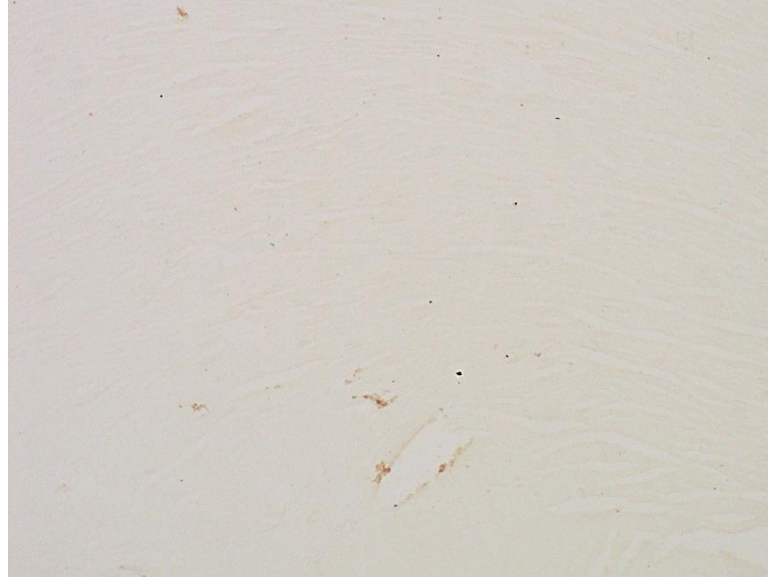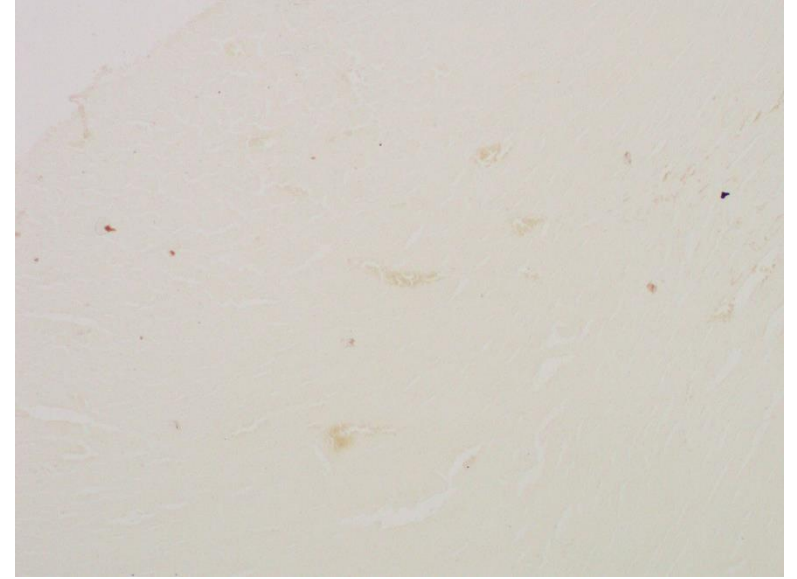

Tamsulosin + ISO

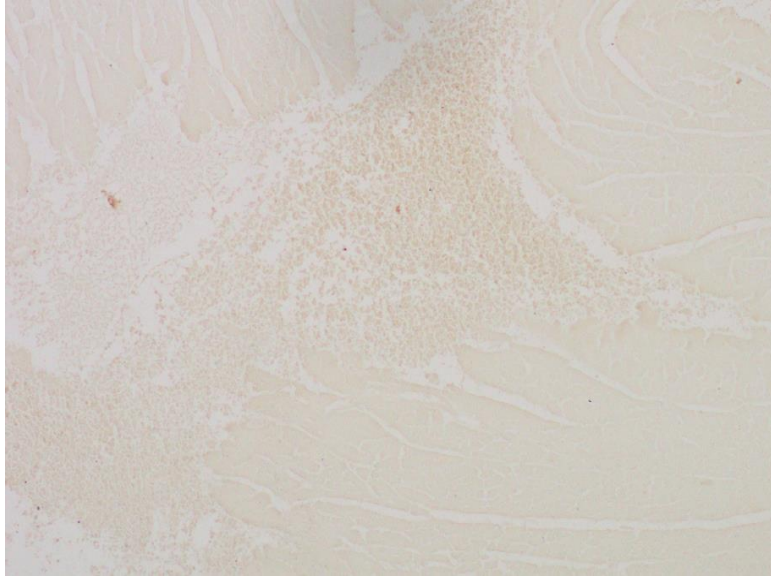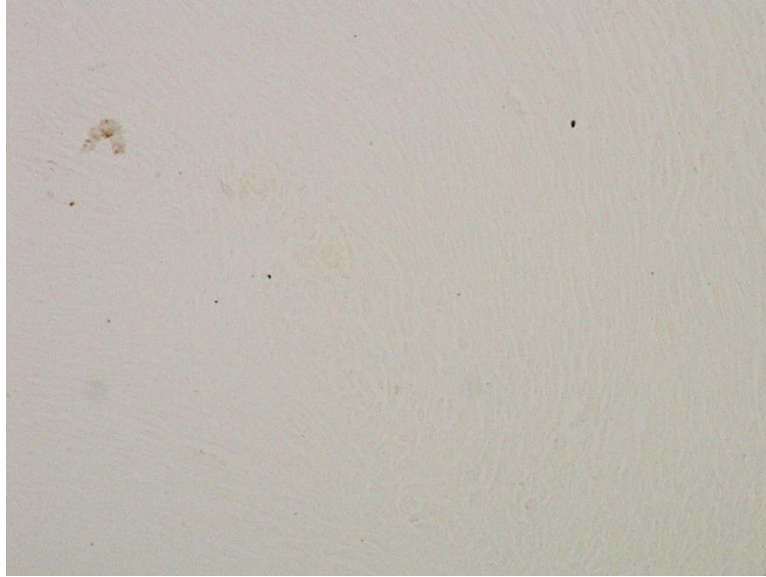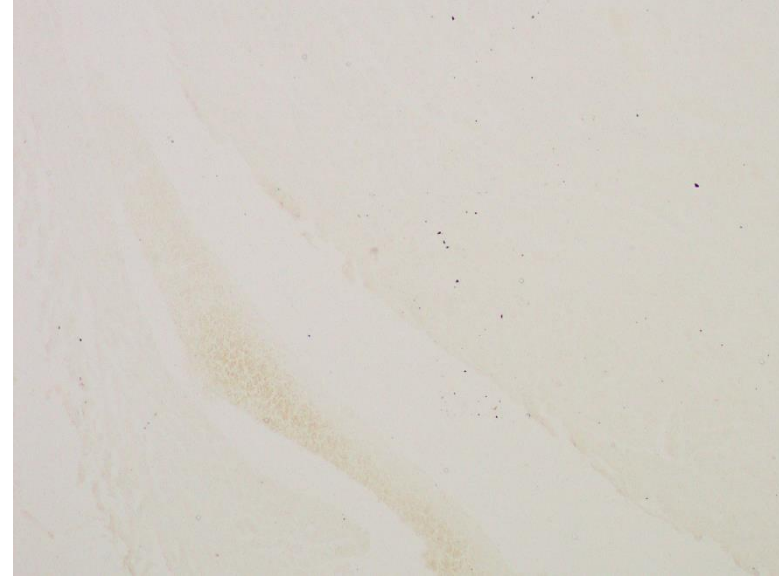

Supplement: Supplementary file 1 — Additional file 1. [file 12872_2023_3188_MOESM1_ESM.zip › 1-original data for figure no. 6. Effect of tamsulosin on collagen III expression in MI induced by ISO. pptx.pdf]

Control

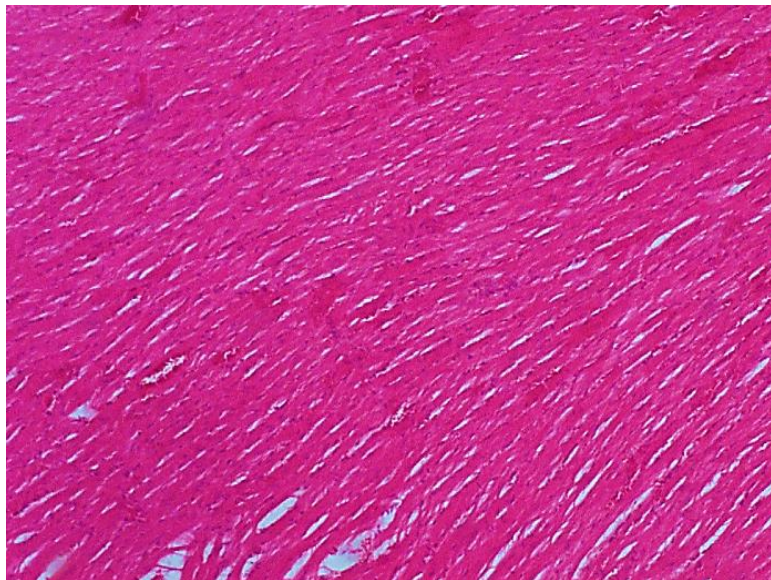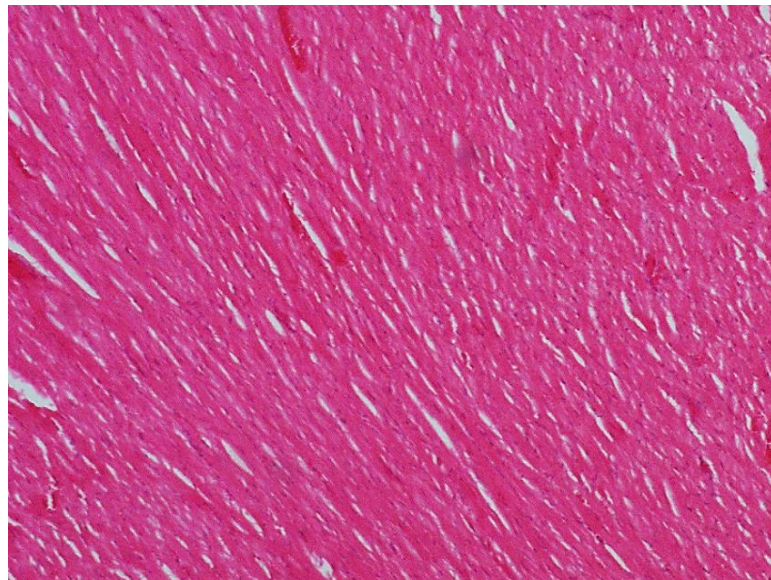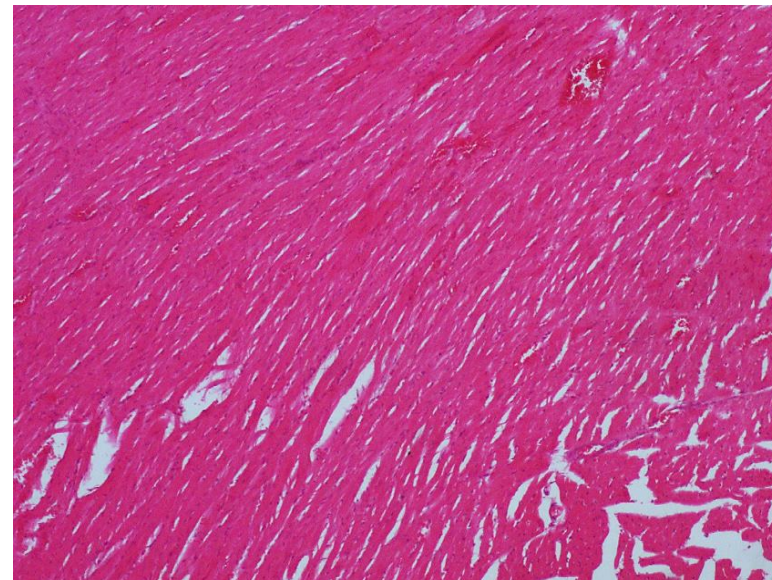

ISO

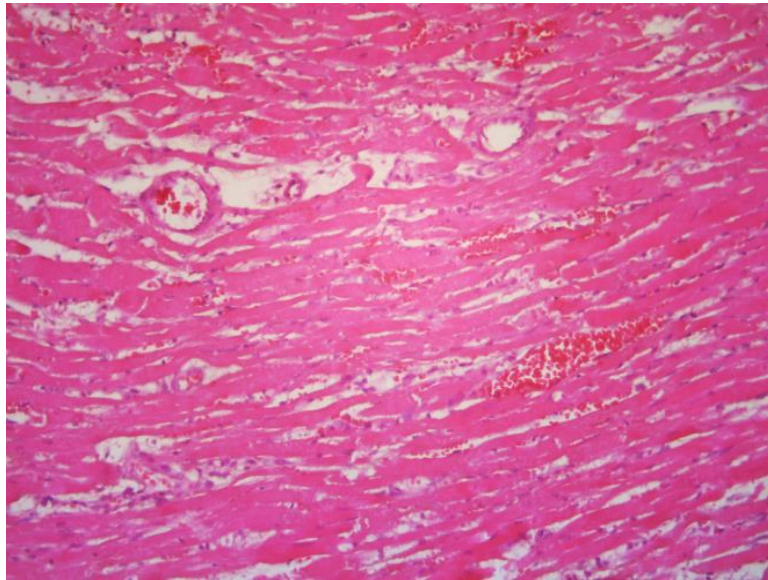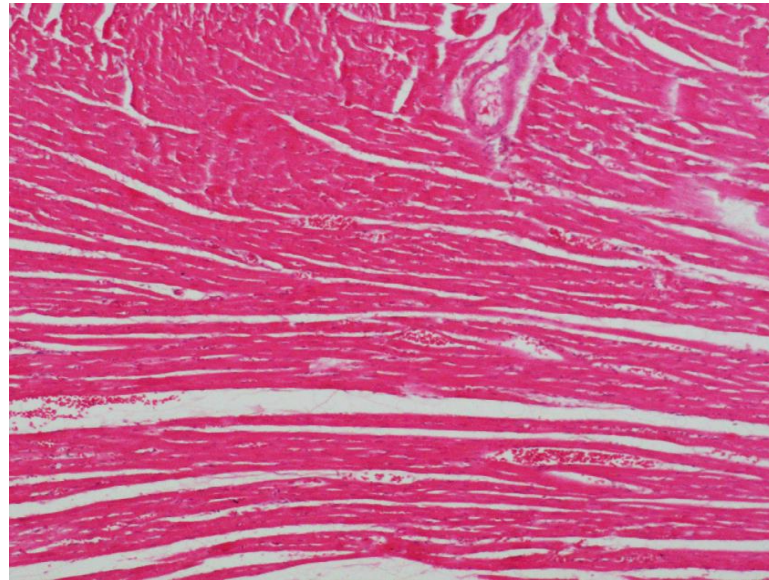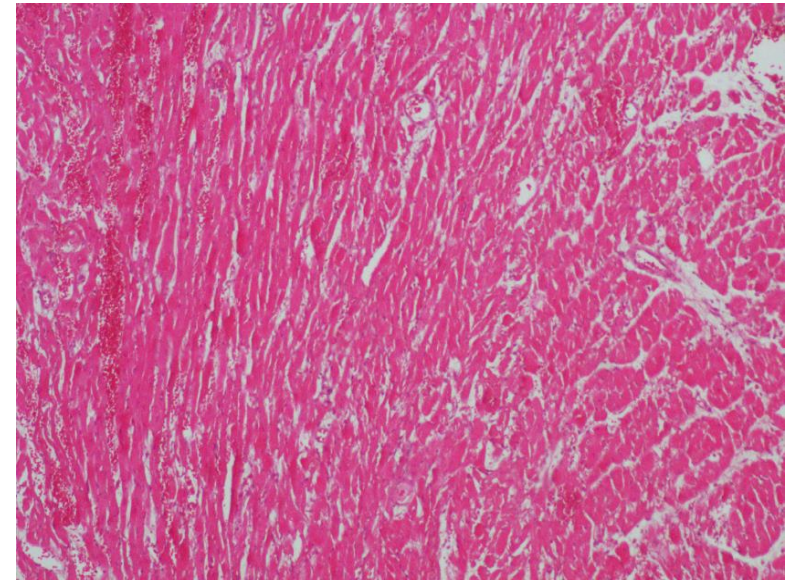

Tamsulosin

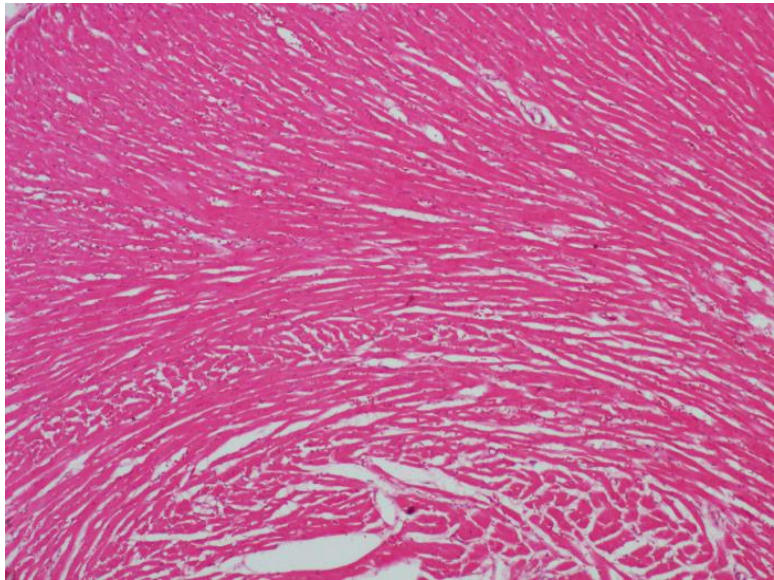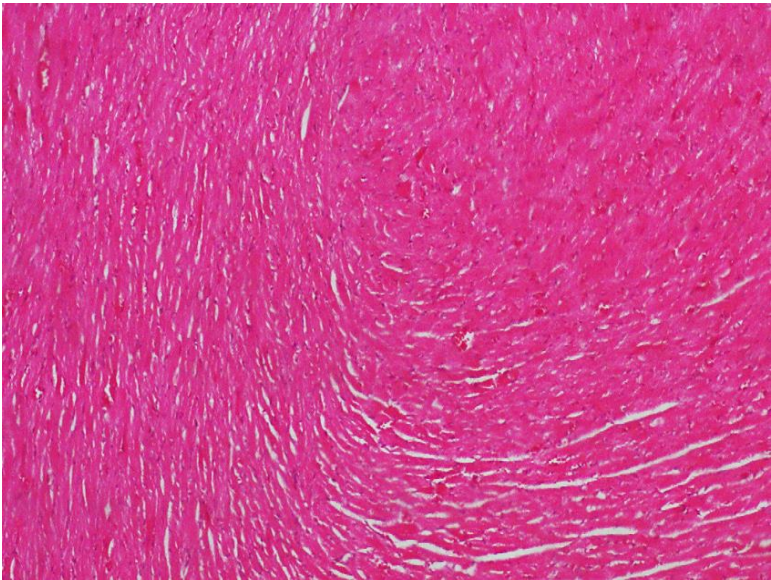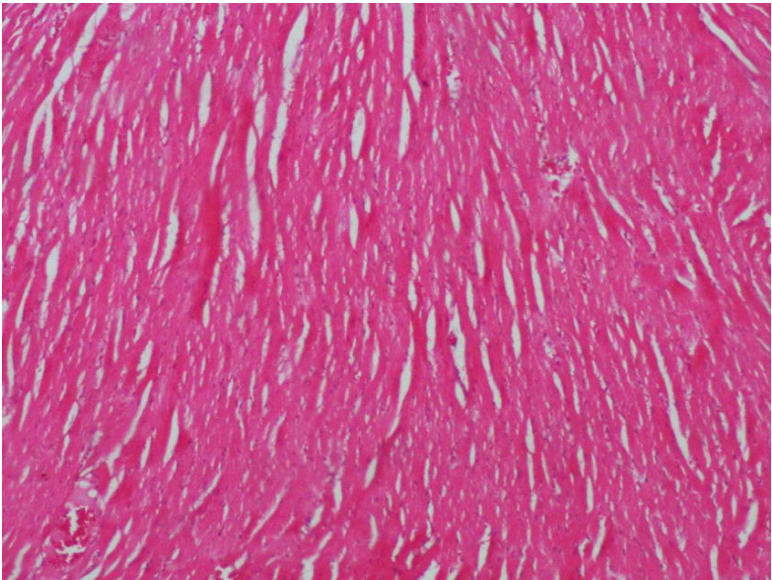

ISO + Tamsulosin

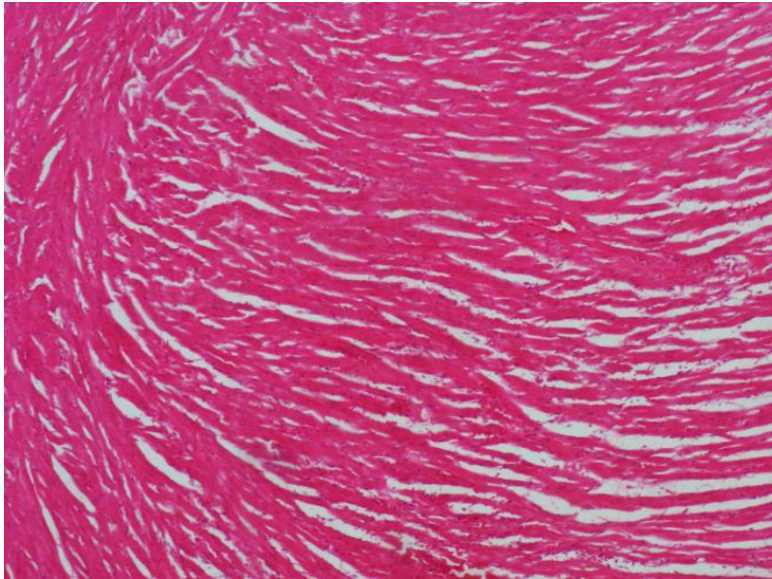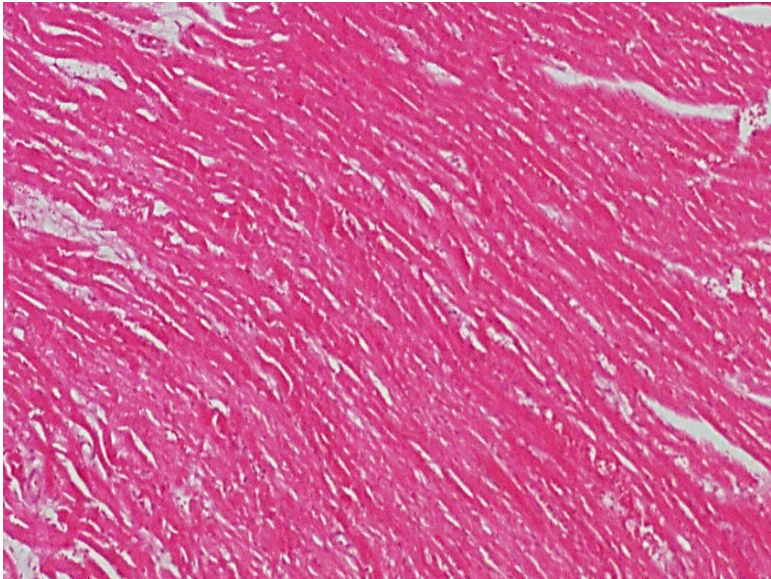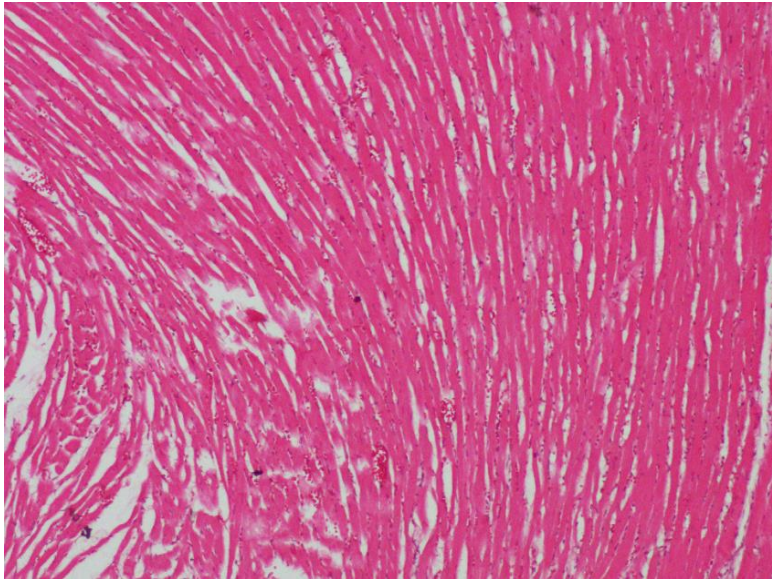

Supplement: Supplementary file 1 — Additional file 1. [file 12872_2023_3188_MOESM1_ESM.zip › original data for figure no. 5. Histological Photomicrographs of rats heart sections stained with H & E (scale bar 10 μm). pptx.pdf]

Control

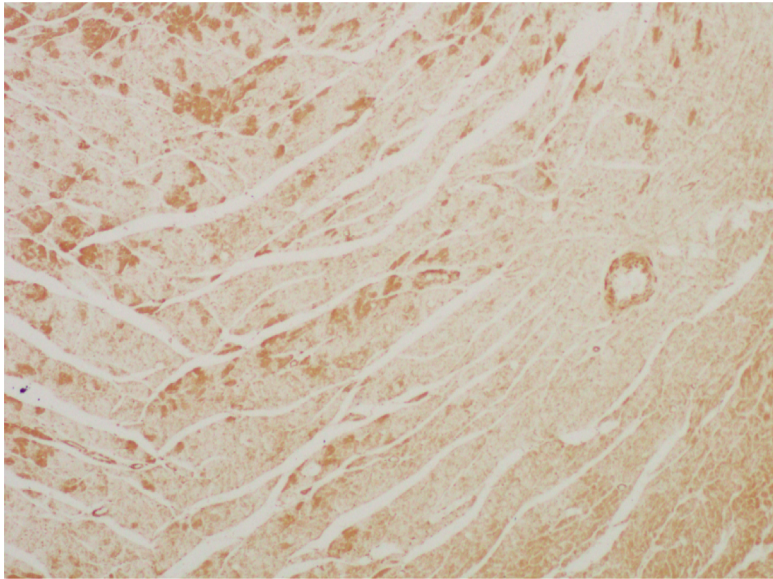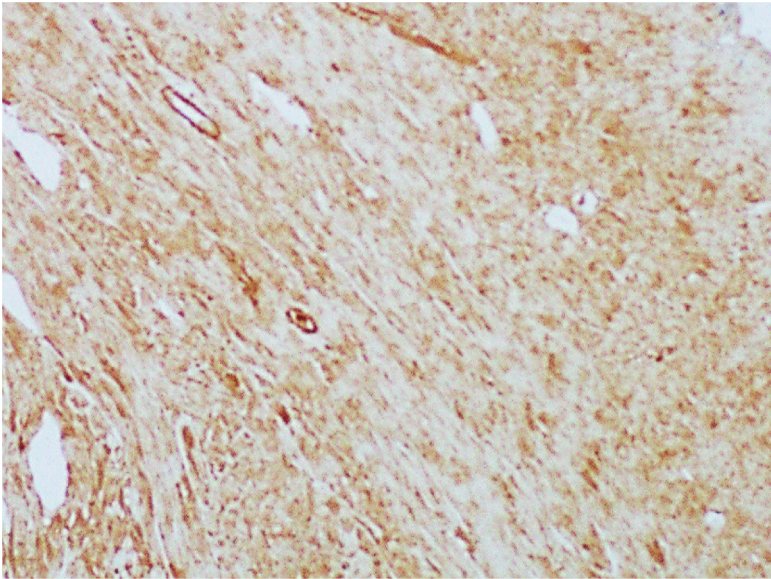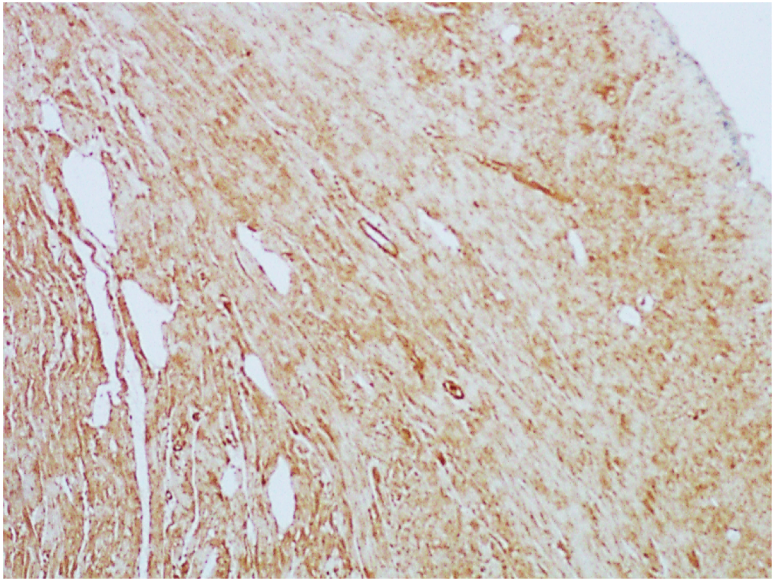

ISO

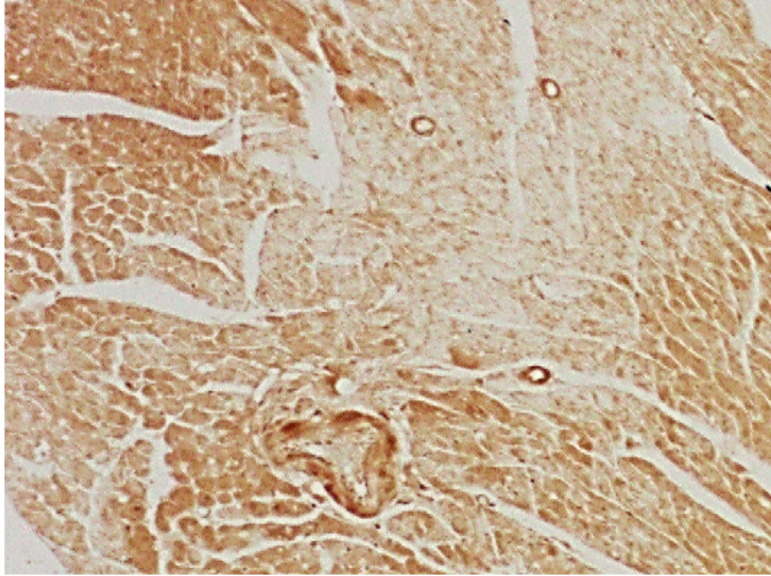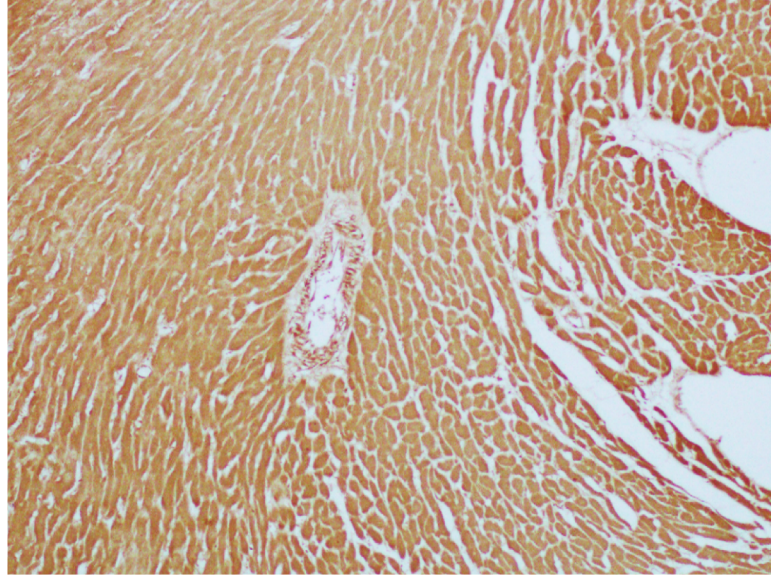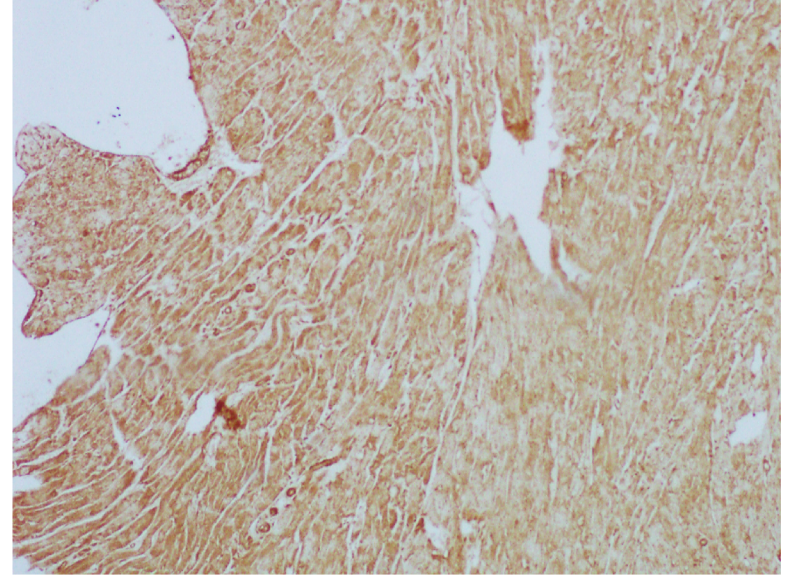

## Tamsulosin

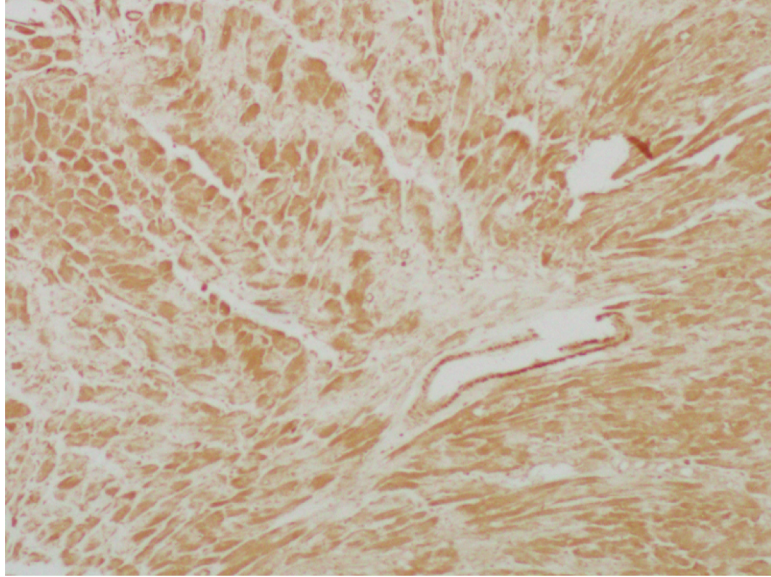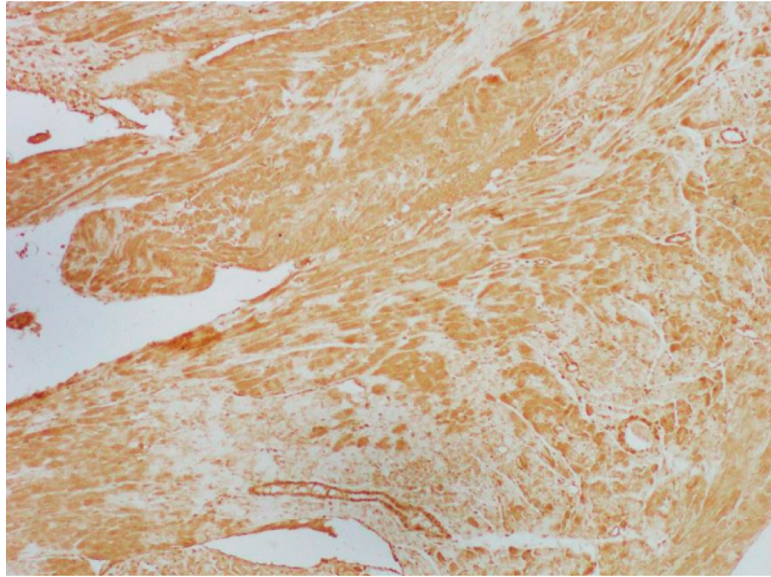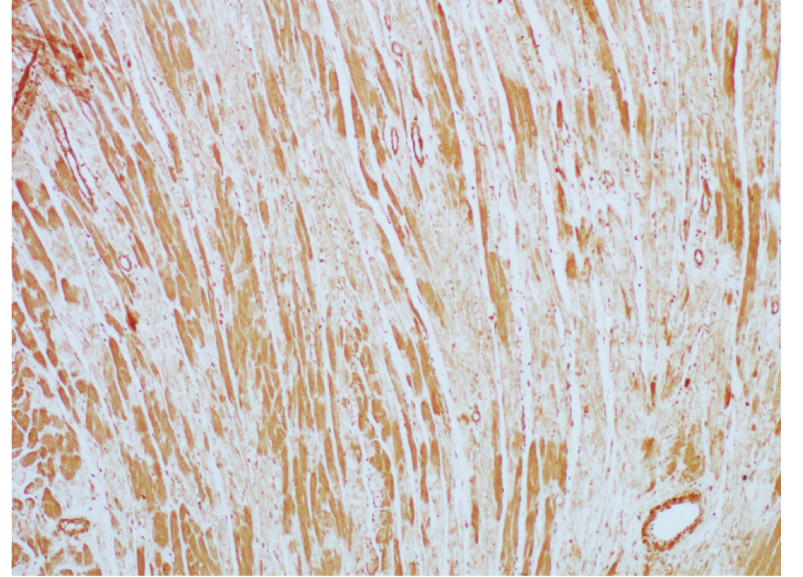

Tamsulosin + ISO

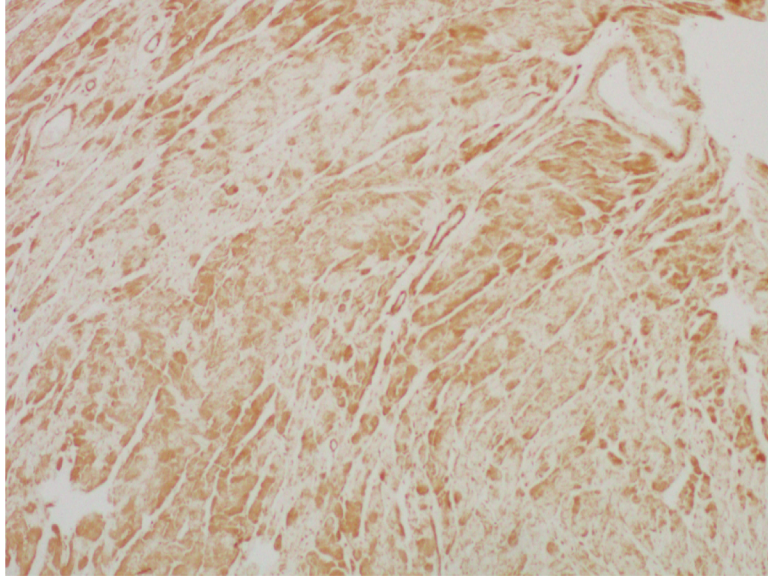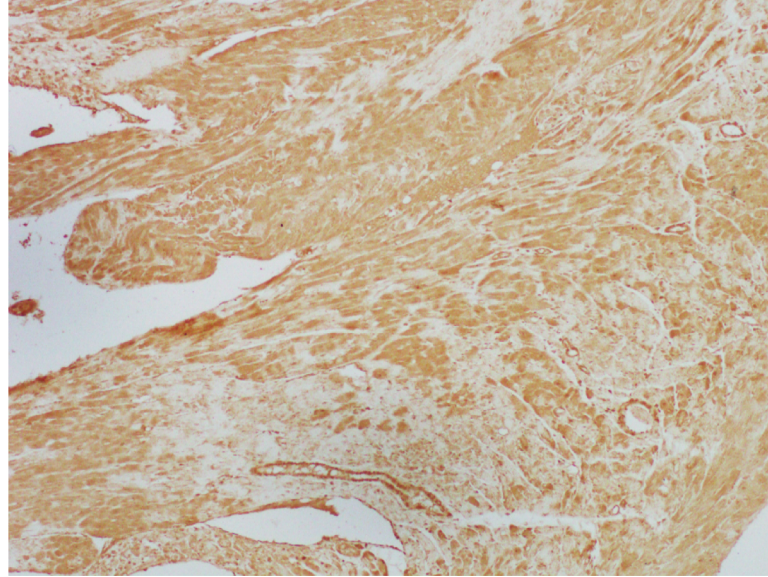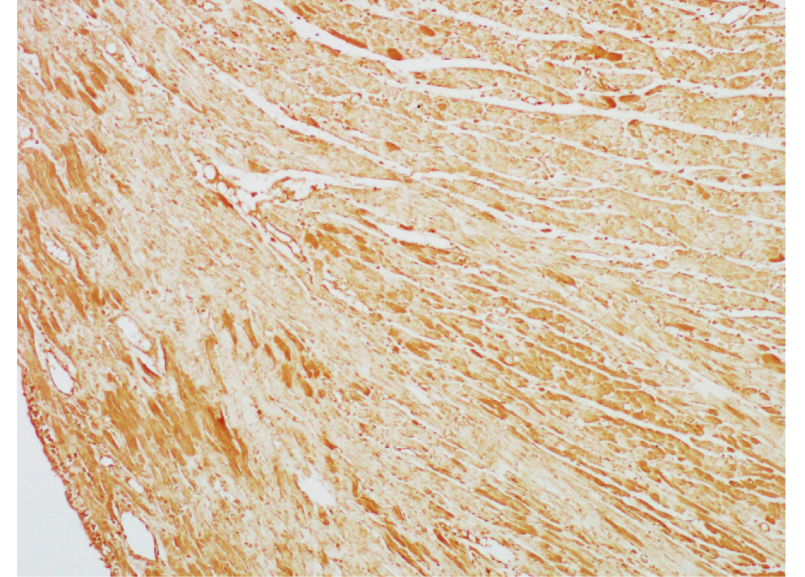

Supplement: Supplementary file 1 — Additional file 1. [file 12872_2023_3188_MOESM1_ESM.zip › original data for figure no. 7. The effect of tamsulosin on Smad 2 3 expression in the myocardial infarcts of ISO-treated rats.pdf]
